# Essential Requirements for Robust Signaling in Hfq Dependent Small RNA Networks

**DOI:** 10.1371/journal.pcbi.1002138

**Published:** 2011-08-18

**Authors:** David N. Adamson, Han N. Lim

**Affiliations:** 1 Biophysics Graduate Group, University of California, Berkeley, California, United States of America; 2 Department of Integrative Biology, University of California, Berkeley, California, United States of America; University of Chicago, United States of America

## Abstract

Bacteria possess networks of small RNAs (sRNAs) that are important for modulating gene expression. At the center of many of these sRNA networks is the Hfq protein. Hfq's role is to quickly match cognate sRNAs and target mRNAs from among a large number of possible combinations and anneal them to form duplexes. Here we show using a kinetic model that Hfq can efficiently and robustly achieve this difficult task by minimizing the sequestration of sRNAs and target mRNAs in Hfq complexes. This sequestration can be reduced by two non-mutually exclusive kinetic mechanisms. The first mechanism involves heterotropic cooperativity (where sRNA and target mRNA binding to Hfq is influenced by other RNAs bound to Hfq); this cooperativity can selectively decrease singly-bound Hfq complexes and ternary complexes with non-cognate sRNA-target mRNA pairs while increasing cognate ternary complexes. The second mechanism relies on frequent RNA dissociation enabling the rapid cycling of sRNAs and target mRNAs among different Hfq complexes; this increases the probability the cognate ternary complex forms before the sRNAs and target mRNAs degrade. We further demonstrate that the performance of sRNAs in isolation is not predictive of their performance within a network. These findings highlight the importance of experimentally characterizing duplex formation in physiologically relevant contexts with multiple RNAs competing for Hfq. The model will provide a valuable framework for guiding and interpreting these experiments.

## Author Summary

Bacteria have small RNAs (sRNAs) which are important modulators of gene expression. Many of these sRNAs require the Hfq protein to mediate their binding to specific target mRNAs which alters the translation and/or degradation of the mRNAs. The Hfq protein has a difficult task; it has to correctly pair cognate sRNAs and target mRNAs from among a large number of possible combinations and anneal them before the RNAs degrade. Furthermore, the process must be robust to changes in the number and types of sRNAs and target mRNAs that are transcribed and changes in the Hfq concentration. Here we show that Hfq can most successfully achieve its task when sRNAs and target mRNAs are not unnecessarily sequestered in Hfq complexes. The cell can accomplish this via cooperative binding of sRNAs and target mRNAs to Hfq and/or by rapid RNA dissociation from Hfq complexes. These findings reveal the requirements for efficient and robust sRNA signaling which are important for understanding the regulation of gene expression in diverse cell processes, for devising strategies that inhibit Hfq activity during pathogenesis and for the rational construction of synthetic circuits.

## Introduction

Small RNAs (sRNAs) regulate a wide variety of pathways in prokaryotes [reviewed in 1]. An important subset of these small RNAs act in *trans* with the aid of the Hfq protein to decrease (“silencing”) or increase (“activation”) the expression of specific target mRNAs. These *trans*-acting, Hfq-dependent sRNAs, which have important roles in the cellular response to stress and the virulence of major pathogens [2], [3], [4], [5], [6], are the focus of this study.

sRNAs typically function by binding to target mRNAs at or near the site of the ribosome binding sequence (RBS) [reviewed in 1]. This results in sRNA-target mRNA duplexes which decrease or less commonly increase the translation of mRNAs. The decreased mRNA translation can be accompanied by an increase in mRNA degradation. The binding between a sRNA and its cognate target mRNA is sequence specific. However, this does not mean that each sRNA can only bind to one target mRNA; a sRNA can act on multiple target mRNAs and a target mRNA can have binding sites for more than one sRNA.

The Hfq protein, which was originally identified as an essential host factor for the replication of the bacteriophage Qβ [7], plays an important role in bringing many sRNAs and target mRNAs together and assisting their annealing. Hfq is a small 11 kDa protein which forms stable cyclic homo-hexamers [8] that have a “Proximal face” and a “Distal face”. The Proximal face binds uridine rich sequences and the distal face binds poly(A) tracts and poly(A-R-N) repeats, where R is a purine nucleotide and N is any nucleotide [9]. Competition studies indicate substantial overlap in the binding sites for sRNAs and target mRNAs on Hfq and they indicate interactions between the RNAs bound to these sites [10], [11]. Hfq also binds to proteins including RNase E [12], polynucleotide phosphorylase (PNPase) [13] and ribosomal subunit S1 [14]. In addition to its role in mediating sRNA activity, Hfq also binds DNA [15], [16] and regulates the degradation of polyadenylated mRNAs [13], [17].

**Figure 1:**
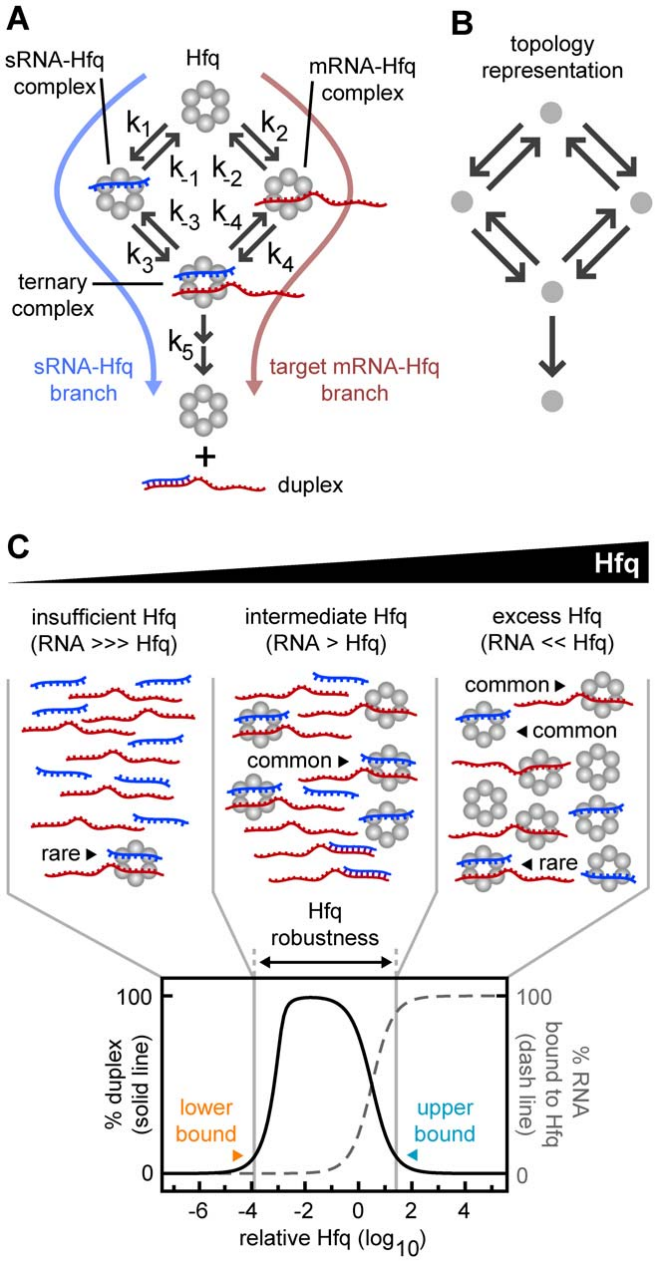
General reaction scheme for Hfq dependent duplex formation. (A) A kinetic model of Hfq-dependent duplex formation showing two paths to duplex formation. The annealing of the sRNA and target mRNA and the release of the duplex from Hfq are treated as a single step (see main text). (B) The kinetic model as a simplified topology representation. (C) Percentage duplex formation and percentage of RNA bound to Hfq at different concentrations of Hfq. The illustration (top) shows mechanistically why insufficient and excess Hfq result in decreased duplex formation. At low concentrations of Hfq, the formation of cognate ternary complexes is limited by the number of Hfq hexamers. At high concentrations of Hfq, the formation of cognate ternary complexes is limited because the probability of a sRNA and its cognate target mRNA binding to the same Hfq hexamer is low. That is, sRNA and target mRNA molecules are sequestered from one another on separate Hfq complexes. The lower and upper bounds indicate the minimum and maximum Hfq concentrations respectively that result in at least 10% duplex formation.

In many sRNA-target mRNA pairs, both members can bind free Hfq. This has been demonstrated in co-immunoprecipitation studies [18], [19], *in vitro* Hfq binding assays [20], [21] and with *in vivo* competition studies [22]. The affinity of the sRNA and the target mRNA for free Hfq in many pairs appears to be comparable [17], [20], [23] (although the RyhB-sodB pair appears to be an exception [21]). Therefore most sRNAs have two potential paths to duplex formation; one where the sRNA binds to free Hfq followed by target mRNA binding (“sRNA-Hfq branch”) and another where the target mRNA binds to free Hfq followed by sRNA binding (“target mRNA-Hfq branch”) (**Figure 1A**).

Once the sRNA and its cognate target mRNA are bound to Hfq (forming a cognate sRNA-Hfq-target mRNA ternary complex), Hfq can promote duplex formation by providing a structure for strand exchange to take place [24], [25] or by acting as a chaperone that alters the sRNA and target mRNA structures to expose sites necessary for annealing [26], [27]. Early *in vitro* evidence suggested that the conformational change in the bound RNAs and annealing was slow and required ten minutes or more to occur [26]. However, more recently it was observed that a partial duplex can form and be released from Hfq with the whole process taking seconds rather than minutes [28]. The basis for the discrepancy between the two studies is unclear but the latter is consistent with *in vivo* experiments which have shown target mRNA silencing occurring within three minutes of sRNA induction [29]. Once the duplex is formed it can be released or remain bound to Hfq while it is degraded or translated (the latter will decrease the availability of free Hfq).

Experimental and theoretical studies have typically focused on duplex formation for individual sRNA-target mRNA pairs in isolation [30], [31]. However, most sRNAs act within a network with dozens of different sRNAs and target mRNAs competing for Hfq [32]. Furthermore, the network is not static but changes its composition of sRNAs, target mRNAs and the amount of Hfq in response to environmental conditions [33], [34], [35]. Because the actions of sRNAs are so interdependent due to their shared need for Hfq, these changes in the network's composition can dramatically alter sRNA activity [22]. In this study we sought to address the fundamental question of how a large network with many ligands (sRNAs and target mRNAs) competing for a single protein (Hfq) can function efficiently and robustly.

In the first part of the study we modeled the kinetics of duplex formation for a single cognate sRNA-target mRNA pair. We identified two non-mutually exclusive mechanisms that can increase the efficiency of sRNA signaling: 1. heterotropic cooperativity for the binding of sRNAs and/or target mRNAs to Hfq (we simply refer to this as “cooperativity”); and 2. frequent RNA dissociation. These mechanisms increase duplex formation by reducing the sequestration of sRNAs and target mRNAs in singly-bound Hfq complexes. In the second part of the study we show the same two mechanisms also promote signaling in sRNA networks with many sRNAs and target mRNAs competing for Hfq. In this case, cooperativity and/or frequent RNA dissociation also decrease the sequestration of sRNAs and target mRNAs in non-cognate ternary complexes (*i.e.* where Hfq is bound by a sRNA and a target mRNA that do not form a cognate duplex). These mechanisms make duplex formation more efficient as well as more robust to changes in the Hfq concentration and the composition of the network.

## Results

### A general model of Hfq kinetics

Current evidence indicates that sRNAs and target mRNAs have separate binding sites on Hfq as well as shared sites. We simply assumed that sRNAs and target mRNAs have separate sites, which provides a conservative estimate of the difficulties faced by Hfq. However, the qualitative findings of this study are still applicable when sRNAs and target mRNAs compete for shared sites on Hfq (see Discussion). There are two possible paths to duplex formation; one where the sRNA binds first to Hfq followed by the target mRNA (“sRNA-Hfq branch”) and another where the target mRNA binds first to Hfq followed by the sRNA (“target mRNA-Hfq branch”) (**Figure 1A**). The reaction scheme, which may be a random order *bi uni* or compulsory order *bi uni* enzymatic reaction [36], can be topologically represented as a graph with weighted, directed arrows indicating the relative magnitude of the rate constant for each reaction (**Figure 1B**).

The complete reaction scheme for a single sRNA-target mRNA pair has three categories of rate constants: 1. association rate constants which describe the binding of sRNAs and target mRNAs to free Hfq or an Hfq complex (k_1_, k_2_, k_3_ and k_4_ with units of concentration^−1^⋅time^−1^); 2. dissociation rate constants for the unbinding of sRNAs and target mRNAs from Hfq complexes (k_−1_, k_−2_, k_−3_ and k_−4_ with units of time^−1^); and 3. a “duplex” rate constant which is an overarching constant for the steps involved in sRNA-target mRNA annealing and the release of the duplex from Hfq (k_5_ with units of time^−1^). While duplexes can rebind Hfq *in vitro* [37], *in vivo* many duplexes are rapidly degraded [reviewed in 1] and therefore we do not include duplex rebinding. The fraction of total target mRNA converted to a specific cognate duplex at steady state is the measured output of the pathway; this output does not depend on whether the sRNA is silencing or activating gene expression.

We varied the rate constants to generate reaction schemes with different topologies. Unless otherwise stated, we kept the production and degradation of the sRNAs and target mRNAs constant and equal (unless otherwise stated) and varied the total Hfq concentration by altering its production (a typical plot is shown in **Figure 1C**). That is, the concentration of Hfq varies relative to the total target mRNA. Therefore when the “relative Hfq” = 1 (*i.e.* 10^0^), it indicates the concentration of Hfq in all forms is equal to the total concentration of target mRNA for the cognate pair being measured (which includes free target mRNA, target mRNA bound to Hfq and target mRNA in duplexes). The minimum and maximum relative Hfq concentrations that permit at least 10% duplex formation, were termed the “lower bound” and the “upper bound” respectively. The logarithmic range of Hfq concentrations over which at least 10% of target mRNA is converted to duplex (*i.e.* the fold-difference between the upper and lower bounds) is termed “Hfq robustness”. Hfq robustness is a useful overall measure of how efficiently the system copes with changes in Hfq, sRNA and target mRNA concentrations and with competition for Hfq.

At the lower bound, the Hfq concentration is insufficient to process the quantity of sRNAs and target mRNAs present (**Figure 1C**). Typically the lower bound is determined by the Hfq recycling rate; that is, the rate at which free Hfq is converted to ternary Hfq complex and then to free duplex and free Hfq. At the upper bound, the sRNA and target mRNA concentrations are low compared to Hfq. Therefore there is a low probability that the sRNA and its cognate target mRNA will bind to the same Hfq hexamer to form the ternary complex, and a high probability they will bind to separate Hfq hexamers to form singly-bound complexes (sRNA-Hfq and target mRNA-Hfq). The upper bound is consequently a measure of the susceptibility of a pathway to sequester sRNAs and target mRNAs in singly-bound Hfq complexes. It has been shown *in vitro* that relatively high Hfq concentrations do indeed increase singly-bound Hfq complexes and reduce duplex formation [20], [23].

To keep the model as simple as possible, the rate constant *β* (equal to 1 unit of time^−1^) for degradation and dilution is the same for all species. While this simplification does not reflect the relative degradation rates in biological systems (*e.g.* sRNAs often have longer half-lives when bound to Hfq), it does not alter the basic qualitative results. This was demonstrated by showing that a 10-fold greater degradation rate constant for the free sRNA compared to the sRNA bound to Hfq had minimal effect on the behavior of the system with several different kinetic schemes (**Figure S1**). The reason the free sRNA degradation rate has a minor effect at high Hfq concentrations is that most of the sRNA is bound to Hfq. At low Hfq concentrations, the free sRNA degradation has minimal effect because there is insufficient Hfq to bind all the sRNA.

### Part 1: Duplex formation for a single sRNA-target mRNA pair

#### Independent binding of sRNAs and target mRNAs to Hfq

**Figure 2:**
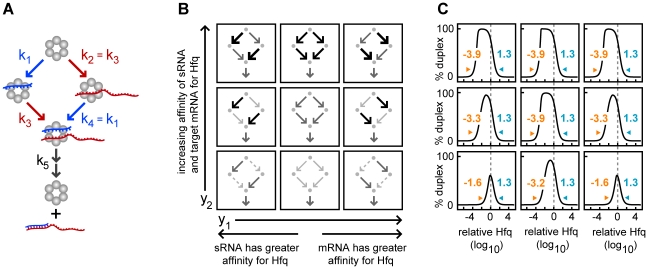
Independent binding of sRNAs and target mRNAs to Hfq. (A) Reaction scheme with independent binding of sRNAs and target mRNAs to free Hfq hexamers and Hfq complexes (k_1_ = k_4_ and k_2_ = k_3_). In this scheme there is no sRNA or target mRNA dissociation. (B) Topological representation of the reaction schemes with low, medium and high values for the y_1_ and y_2_ parameters. y_1_ determines the relative affinity of target mRNAs and sRNAs for Hfq [y_1_≡((k_2_⋅k_3_)/(k_1_⋅k_4_))^1/2^] and it has values of 10^−4^, 10^0^ and 10^4^ (unitless) in the simulations. y_2_ determines the overall magnitude of the association rate constants for the sRNAs and target mRNAs [y_2_≡(k_1_⋅k_2_⋅k_3_⋅k_4_)^1/4^] and it has values of 10^0.5^, 10^2.5^ and 10^4.5^ concentration^−1^⋅time^−1^ in the simulations. The relative magnitude of the kinetic parameters is represented graphically by the weight of the arrows. (C) Percentage duplex formation at different concentrations of Hfq. Each panel corresponds to the reaction scheme shown in the previous panel at the same position. The grey dash line indicates a 1∶1 ratio of [total target mRNA] to [Hfq], where the [total target mRNA]≡[T]+[HT]+[HST]+[D] and [T], [HT], [HST] and [D] are the concentrations of free target mRNA, target mRNA-Hfq complex, cognate ternary complex and duplex respectively. Yellow values indicate the “lower bound” while blue values indicate the “upper bound” as defined in the main text.

To understand the basic behavior of Hfq mediated duplex formation, we began with a highly simplified model with few kinetic degrees of freedom and gradually introduced additional parameters. We first examined duplex formation in the absence of RNA dissociation (k_−1_ = k_−2_ = k_−3_ = k_−4_ = 0) and with independent binding (**Figure 2A**). Independent binding means the probability that a sRNA or target mRNA binds to Hfq does not depend on whether the Hfq is already bound (*i.e.* k_1_ = k_4_ and k_2_ = k_3_). With independent binding, a system with a fixed value for duplex annealing and release (k_5_) has only two free parameters. We reparameterized them to yield two new parameters, y_1_ and y_2_ (**Figure 2B, C**). y_1_ determines the relative affinity of sRNAs and target mRNAs for Hfq [y_1_≡((k_2_⋅k_3_)/(k_1_⋅k_4_))^1/2^; unitless]. y_2_ specifies the overall magnitude of the sRNA and target mRNA binding [y_2_≡(k_1_⋅k_2_⋅k_3_⋅k_4_)^1/4^; units of concentration^−1^⋅time^−1^].

We selected low, intermediate and high values for y_1_ and for y_2_ resulting in nine representative reaction schemes [y_1_ = 10^−4^, 10^0^ and 10^4^; y_2_ = 10^0.5^, 10^2.5^ and 10^4.5^ concentration^−1^⋅time^−1^]. For each reaction scheme, the percentage of the target mRNA converted to duplex was measured at varying Hfq concentrations (**Figure 2C**). Duplex formation was shown to require less Hfq when sRNAs and target mRNAs bind more rapidly to Hfq (*i.e.* increasing y_2_ decreases the lower bound). The lower bound decreases, because with all other factors being equal, increasing RNA binding to Hfq increases Hfq recycling. The maximum concentration of Hfq at which duplex formation occurred (*i.e.* the upper bound) was invariant to y_1_ and y_2_. That is, sRNA and target mRNA sequestration in singly-bound Hfq complexes is unaffected by the kinetic parameters in a system with independent binding and without RNA dissociation.

#### Cooperative binding and dissociation reactions can increase the efficiency and robustness of duplex formation

Heterotropic cooperativity, which we simplify to “cooperativity”, exists when the binding and unbinding of a sRNA or target mRNA to a given Hfq hexamer is not independent but depends upon whether that Hfq hexamer is already bound to an RNA. Heterotropic cooperativity could be due to an allosteric change in the Hfq hexamer or a direct or indirect interaction between the bound sRNA and target mRNA. “Positive cooperativity” occurs when the affinity of a sRNA or target mRNA is greater for the singly-bound Hfq complex than for the free Hfq and “negative cooperativity” occurs when the reverse is true. When positive and negative cooperativity arise because the *binding* of a sRNA and target mRNA to the Hfq is altered by the presence of a bound RNA then we use the more specific terms “positive cooperative association” and “negative cooperative association” respectively. Whereas when positive and negative cooperativity occur because the *unbinding* of a sRNA and a target mRNA from Hfq is altered by the presence of a bound RNA then we use the terms “positive cooperative dissociation” and “negative cooperative dissociation” respectively.

**Figure 3:**
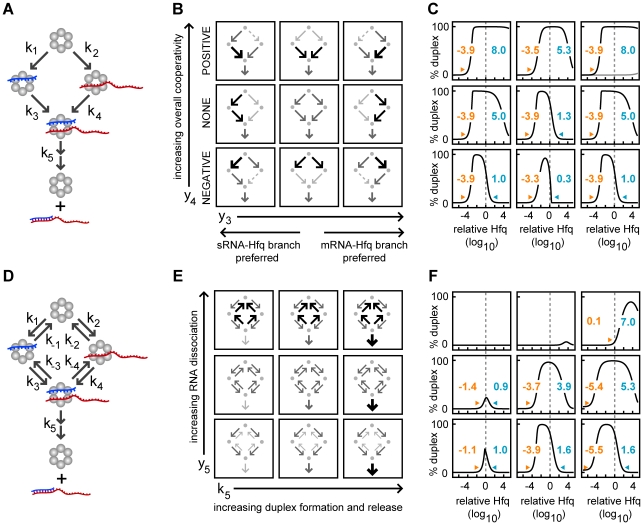
Cooperative binding and dissociation reactions can increase the efficiency and robustness of duplex formation. (A) Reaction scheme without sRNA and target mRNA dissociation. (B) Topological representation of the cooperative reaction schemes with low, medium and high values for y_3_ and y_4_. y_3_ determines whether the association rate constants favor the sRNA-Hfq branch or the target mRNA-Hfq branch [y_3_≡((k_2_⋅k_4_)/(k_1_⋅k_3_))^1/2^] and it has values of 10^−4^, 10^0^ and 10^4^ (unitless) in the simulations. y_4_ biases the system from negative to positive cooperative association [y_4_≡((k_3_⋅k_4_)/(k_1_⋅k_2_))^1/2^] and it has values of 10^−4^, 10^0^ and 10^4^ (unitless) in the simulations. The relative magnitude of the kinetic parameters is represented graphically by the weight of the arrows. (C) As described for **Figure 2C**. (D) Reaction scheme for duplex formation with association and dissociation reactions that are independent. (E) Topological representation of the reaction schemes with low, medium and high values for the k_5_ and y_5_ parameters. k_5_ is the overall rate of duplex formation and release with values of 10^0^, 10^3^ and 10^6^ time^−1^ in the simulations. y_5_ determines the overall magnitude of the dissociation rate constants for the sRNA and target mRNA [y_5_≡(k_−1_⋅k_−2_⋅k_−3_⋅k_−4_)^1/4^] and it has values of 10^0^, 10^4^ and 10^8^ time^−1^ in the simulations. (F) As described for **Figure 2C**.

We relaxed our assumption of independent binding and created two parameters, y_3_ and y_4_, to comprehensively explore the effect of positive and negative cooperative association while keeping fixed the relative affinity of sRNAs and target mRNAs for Hfq (y_1_) and the total magnitude of the association rate constants (y_2_) (**Figure 3A–C**). y_3_ tunes the relative cooperative association of the sRNA compared to that of the target mRNA which alters the bias for the sRNA-Hfq branch and the target mRNA-Hfq branch [y_3_≡(k_2_⋅k_4_)/(k_1_⋅k_3_))^1/2^; unitless] (**Figure 3B**). When y_3_<1, cooperative association is greater for the target mRNA than the sRNA (*i.e.* k_3_/k_2_>k_4_/k_1_) resulting in a bias for the sRNA-Hfq branch. Alternatively when y_3_>1, cooperative association is greater for the sRNA than for the target mRNA (*i.e.* k_4_/k_1_>k_3_/k_2_) resulting in a bias for the target mRNA-Hfq branch. When y_3_ = 1, sRNA and target mRNA cooperative association are equal and therefore duplex formation occurs equally via both branches. y_4_ determines whether the RNA is more or less likely to bind to Hfq after its partner has bound (y_4_>1 and y_4_<1 respectively) [y_4_≡((k_3_⋅k_4_)/(k_1_⋅k_2_))^1/2^; unitless]. When an RNA is more likely to bind to Hfq after its partner has bound we term this positive cooperative association (k_1_<k_4_ and/or k_2_<k_3_). When an RNA is less likely to bind after its partner has bound we term this negative cooperative association (k_1_>k_4_ and/or k_2_>k_3_).

We selected low, intermediate and high values for y_3_ and y_4_, resulting in nine representative reaction schemes [y_3_ = 10^−4^, 10^0^ and 10^4^; y_4_ = 10^−4^, 10^0^ and 10^4^] (**Figure 3B, C**). Our analysis shows that a bias for one RNA binding order (*i.e.* sRNA-Hfq branch or target mRNA-Hfq branch) diminishes sRNA and target mRNA sequestration in singly-bound Hfq complexes which increases the upper bound (compare left or right columns with center column in **Figure 3C**). Positive cooperative association (y_4_>1) also alleviates the sequestration of sRNAs and target mRNAs in singly-bound complexes at high Hfq concentrations, while negative cooperative association (y_4_<1) exacerbates it (compare upper bound in the top and bottom rows in **Figure 3C**). In summary, rate constants that result in a compulsory order of RNA binding (*i.e.* a strong bias for the sRNA-Hfq branch or the target mRNA-Hfq branch) and/or positive cooperative association increase the maximum Hfq concentration at which duplex formation can occur thereby increasing Hfq robustness.

#### Frequent RNA dissociation increases the efficiency and robustness of duplex formation

We next incorporated RNA dissociation, governed by the rate constants k_−1_, k_−2_, k_−3_, k_−4_, into a model with independent RNA binding and balanced affinity of sRNAs and target mRNAs for Hfq (**Figure 3D**). In the context of the reverse reactions, these criteria imply that k_1_ = k_2_ = k_3_ = k_4_ and k_−1_ = k_−2_ = k_−3_ = k_−4_. The parameter y_5_ determines the overall probability of non-duplex RNA dissociating from Hfq [y_5_≡(k_−1_⋅k_−2_⋅k_−3_⋅k_−4_)^1/4^, units of time^−1^]. Because the dissociation of non-duplex RNA from Hfq constitutes backtracking along the paths to duplex formation, we vary y_5_ in conjunction with k_5_ (the rate constant for duplex annealing and release) which opposes its action. To systematically explore the impact of these competing effects, we selected low, intermediate and high values for y_5_ and for k_5_, resulting in nine reaction schemes [y_5_ = 10^0^, 10^4^ and 10^8^ time^−1^; k_5_ = 10^0^, 10^3^ and 10^6^ time^−1^] (**Figure 3E, F**).

Increasing duplex annealing and release (k_5_) increases Hfq recycling and reduces RNA sequestration in singly-bound complexes. This decreases the minimum Hfq concentration and increases the maximum Hfq concentration for duplex formation leading to increased Hfq robustness (**Figure 3E, F**). Moderately increasing RNA dissociation such that y_5_ is greater than the RNA degradation rate can diminish the sequestration of sRNA and target mRNA molecules in singly-bound Hfq complexes resulting in an increased upper bound (**Figure 3F**). The increased dissociation of sRNAs and target mRNAs from Hfq complexes enables them to bind multiple Hfq hexamers before they degrade which increases the likelihood that they will encounter their partner on the same Hfq complex (*i.e.* forming a cognate ternary complex). However, the increased RNA dissociation also diminishes the overall RNA affinity for Hfq and therefore higher Hfq concentrations are needed for duplex formation (increased lower bound in **Figure 3F**). If RNA dissociation is too great then duplex formation is prevented (upper left panel, **Figure 3F**).

#### Cooperativity combined with frequent RNA dissociation can result in greater robustness than either mechanism alone

**Figure 4:**
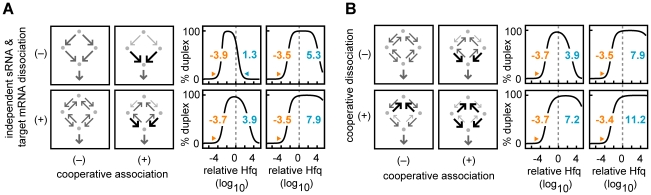
Cooperative binding and dissociation synergistically promote duplex formation. The combination of cooperative association plus independent dissociation reactions (A) or cooperative association plus cooperative dissociation (B). (A) Duplex formation was simulated with or without independent, RNA dissociation (k_−1_ = k_−2_ = k_−3_ = k_−4_ = y_5_ = 10^4^ or 0 time^−1^; lower and upper panels respectively) and with or without cooperative association (y_4_ = 10^4^ or 10^0^ unitless, right and left panels respectively). The four plots on the right of this panel are as described in **Figure 2C**. (B) Duplex formation was simulated with or without cooperative dissociation [y_6_≡((k_−1_⋅k_−2_)/(k_−3_⋅k_−4_))^1/2^ and it has values of 10^4^ or 10^0^; lower and upper panels respectively] and with or without cooperative association [y_4_ = 10^4^ or 10^0^ unitless; right and left panels respectively]. The four plots on the right of this panel are as described in **Figure 2C**.

We next demonstrated that cooperativity and RNA dissociation, which promote duplex formation via two different mechanisms, can have synergistic effects. We first showed that positive cooperative association plus *independent* unbinding of sRNAs and target mRNAs from Hfq complexes reduces singly-bound Hfq complexes more than either mechanism alone (upper bound increases; **Figure 4A**). We then examined the effects of positive cooperativity in the dissociation reactions (*i.e.* positive cooperative dissociation as defined earlier) which acts to relatively increase dissociation from singly-bound Hfq complexes compared with dissociation from Hfq ternary complexes (k_−4_<k_−1_ and/or k_−3_<k_−2_) also promotes Hfq robustness (upper bound increases, **Figure 4B**). Cooperativity in the dissociation reactions is quantified by the unitless metric y_6_≡((k_−1_⋅k_−2_)/(k_−3_⋅k_−4_))^1/2^; y_6_>1 indicates positive cooperativity while y_6_<1 indicates negative cooperativity. The combination of positive cooperative association plus positive cooperative dissociation further reduces sequestration in singly-bound Hfq complexes (**Figure 4B**).

### Part 2: Duplex formation in the sRNA network

#### General reaction scheme for duplex formation in a network with multiple sRNAs and target mRNAs

**Figure 5:**
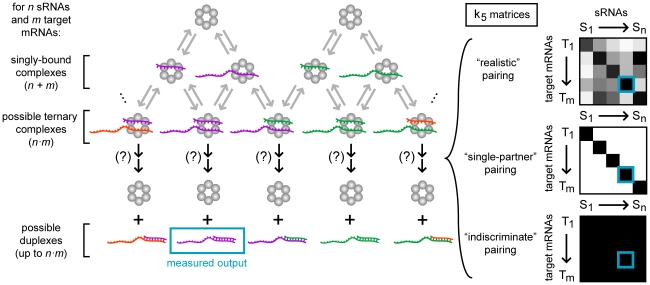
General reaction scheme for duplex formation in a network with multiple sRNAs and target mRNAs. In a network with multiple sRNAs and target mRNAs many types of Hfq ternary complexes are possible. If these ternary complexes cannot form duplexes they are termed non-cognate ternary complexes. In other words, k_5_∼0 for a non-cognate ternary complex and k_5_>>0 for a cognate ternary complex. The k_5_ values that specify whether a ternary complex is cognate or non-cognate can be represented in a *n*×*m* matrix for *n* sRNAs and *m* target mRNAs with the darkness of the shading representing the magnitude of k_5_. Three examples of this k_5_ matrix are shown (see main text): 1. a “mock” native sRNA network (upper right panel); 2. a simplified system where each sRNA and target mRNA has only one specific partner (middle right panel); and 3. a simplified system where each sRNA and target mRNA can indiscriminately form duplexes with any other target mRNA or sRNA respectively (lower, right panel). For subsequent plots, we stress that “relative Hfq” = 1 (*i.e.* 10^0^) indicates the concentration of Hfq in all forms is equal to the total concentration of target mRNA for the cognate pair being measured (as opposed to the total concentration of all the target mRNAs in the network).

In a network, we need to consider that sRNAs and target mRNAs can bind in multiple combinations to Hfq resulting in many types of ternary complexes [reviewed in 1] (**Figure 5**). Therefore we categorized ternary complexes as cognate or non-cognate according to whether the bound sRNAs and target mRNAs can form a duplex or not. It is possible for any given sRNA or target mRNA to have several cognate RNA partners. By definition, if a ternary complex is non-cognate then k_5_∼0 and if a ternary complex is cognate then k_5_>>0.

In any real biological system k_5_ will have a range of values for every possible ternary complex that can form and therefore the distinction between cognate and non-cognate may not be clear. That is, in a real network there will be many sRNAs and target mRNAs that can form duplexes with more than one type of target RNA or sRNA respectively (as stated above). Furthermore, each sRNA or target mRNA may form these different types of duplexes at different rates. The k_5_ value, which determines the rate of duplex annealing and release, can be represented in a “k_5_ matrix” for each possible sRNA-target mRNA pairing on Hfq (a “mock” example of a k_5_ matrix for a “real” system is shown in the upper right panel, **Figure 5**; darker shading indicates higher magnitudes of k_5_ with white representing zero).

**Figure 6:**
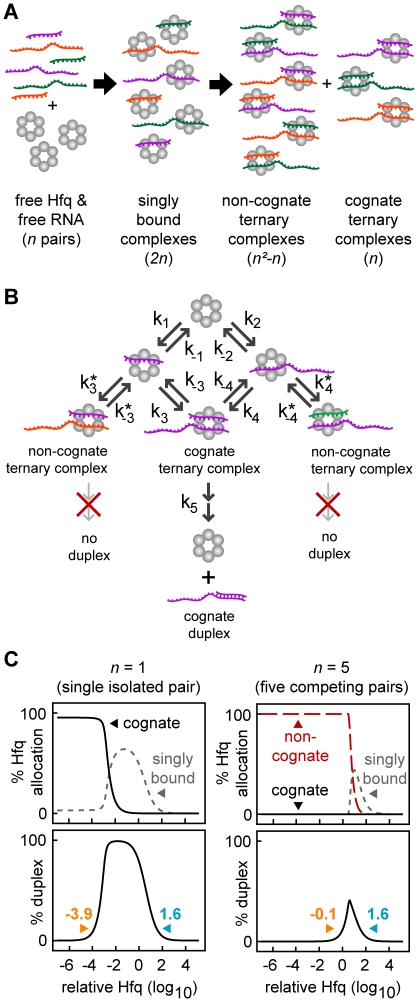
Non-cognate ternary complexes decrease the efficiency and robustness of duplex formation. (A) Schematic showing the possible Hfq complexes formed by three sRNAs and three target mRNAs with single partner pairing. The number of possible types of Hfq complexes that can form with *n* sRNA-target mRNA pairs if both the sRNA and the target mRNA in each pair can bind to Hfq hexamers and there is only one cognate partner for each sRNA and target mRNA. (B) Schematic showing the kinetics of duplex formation for a single cognate sRNA-target mRNA pair in the presence of multiple sRNAs and target mRNAs competing for Hfq. Non-cognate ternary complexes form but by definition do not result in duplexes. The rate constants k*_3_ and k*_4_ specify the association of the target mRNA and sRNA respectively to a sRNA-Hfq and target mRNA-Hfq complex resulting in the formation of a non-cognate ternary complex. The rate constants k*_−3_ and k*_−4_ specify the dissociation of the target mRNA and sRNA respectively from the non-cognate ternary complex. (C) The percentage allocation of Hfq and percentage duplex formed for a sRNA-target mRNA pair in isolation (left panels) and in a network with five sRNA-target mRNA pairs competing equally for Hfq (right panels). In these simulations, all sRNA-target mRNA pairs have independent RNA binding and unbinding. Yellow and blue values indicate the lower and upper bounds respectively.

We reduced the complexity of the system by adding the constraint that every sRNA and target mRNA behaves identically and the number of sRNAs (*n*) is equal to the number of target mRNAs (*m*) (*i.e. n* = *m*) unless otherwise stated. We examined two extreme cases of this simplified system. In the first case, each sRNA and target mRNA has only one specific partner. That is, sRNA_i_ forms a duplex with mRNA_j_ if and only if *i* = *j* (by definition this means that k_5(i,j)_>>0 if and only if *i* = *j* in the k_5_ matrix) (middle right panel, **Figure 5**). In the second case, each sRNA and target mRNA can indiscriminately form duplexes with any other target mRNA or sRNA respectively. That is, sRNA_i_ forms duplex with mRNA_j_ for all possible pairs *i* and *j* (by definition this means k_5(i,j)_>>0, for all values of *i* and *j* in the k_5_ matrix) (lower right panel, **Figure 5**). We examined the first case where each sRNA and target mRNA has exactly one cognate partner in **Figures 6** **–** [Fig pcbi-1002138-g007] [Fig pcbi-1002138-g008] [Fig pcbi-1002138-g009] **10** and **Figure 12** and the second case where sRNAs and target mRNAs can form duplexes in all possible combinations in **Figure 10**.

**Figure 7 pcbi-1002138-g007:**
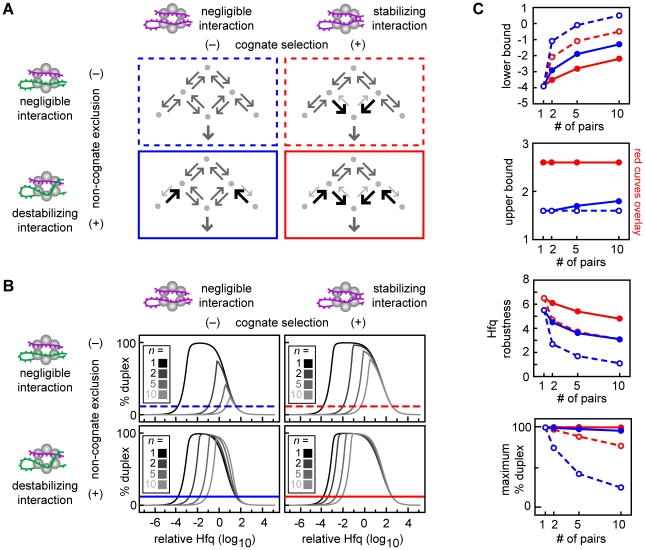
Decreasing non-cognate ternary complexes by cooperativity increases the efficiency and robustness of duplex formation. (A) Simplified topology representations showing the reaction schemes with or without cognate selection and with or without non-cognate exclusion. Each RNA can form duplexes with only one specific partner. Cognate selection occurs when the rate constants increase the formation and/or the stability of the cognate ternary complex such that the unitless ratio y_7_<1 (right panels). Non-cognate exclusion when rate constants decrease the formation and/or the stability of the non-cognate ternary complex such that the unitless ratio y_8_>1 (lower panels). The color and style of the border surrounding each topology indicates the properties of the reaction scheme. Dash blue is independent binding (y_7_ = 1; y_8_ = 1); solid blue is non-cognate exclusion (y_7_ = 1; y_8_ = 10^1^); dash red is cognate selection (y_7_ = 10^−1^; y_8_ = 1); solid red is cognate selection plus non-cognate exclusion (y_7_ = 10^−1^; y_8_ = 10^1^). (B) Percentage duplex formed in sRNA networks with *n* identical sRNA-target mRNA pairs having the reaction scheme shown in the corresponding panel in (A). The horizontal line indicates 10% duplex formation which defines the upper and lower bounds as previously described. (C) The lower bound, upper bound, Hfq robustness and maximum percentage duplex for each kinetic scenario described in (A). The color and style of each curve indicates the corresponding topology shown in (A).

**Figure 8 pcbi-1002138-g008:**
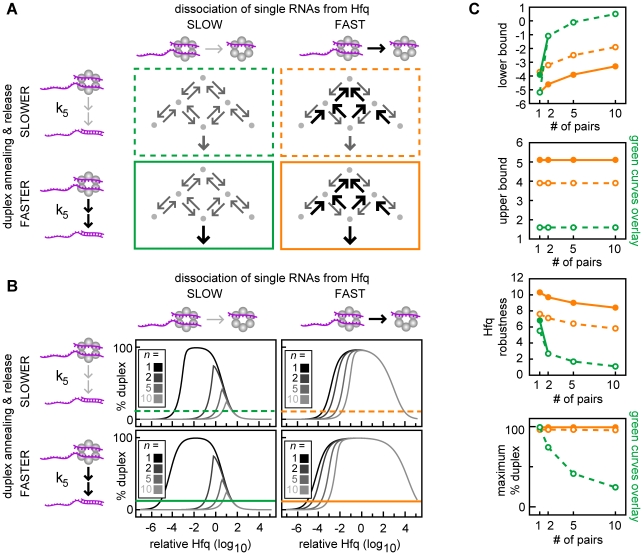
Increasing RNA dissociation increases the efficiency and robustness of duplex formation in networks. (A) Simplified topology representations showing the reaction schemes with and without increased RNA dissociation and with and without increased duplex annealing. Each RNA can form duplexes with only one specific partner. The parameter for duplex annealing and release (k_5_) has values of 10^3^ and 10^6^ time^−1^ for these simulations (upper and lower panels respectively). RNA dissociation from singly-bound Hfq complexes (quantified by y_9_) is non-cooperative and is equal for the sRNA and mRNA within these topologies (*i.e.* k_−1_ = k_−2_ = k_−3_ = k_−4_ = k*_−3_ = k*_−4_ = y_9_ = 10^0^ and 10^4^ time^−1^; left and right panels respectively). The color and style of the border surrounding each topology indicates the properties of the reaction scheme. Dash green serves as a basis for comparison and is identical to dash blue from **Figure 7** (k_5_ = 10^3^; y_9_ = 10^0^); solid green indicates increased duplex annealing and release (k_5_ = 10^6^; y_9_ = 10^0^); dash yellow indicates increased RNA dissociation (k_5_ = 10^3^; y_9_ = 10^4^); and solid yellow indicates both increased RNA dissociation and increased duplex annealing and release (k_5_ = 10^6^; y_9_ = 10^4^). (B) Percentage duplex formed in sRNA networks with *n* identical sRNA-target mRNA pairs with the reaction scheme shown in the corresponding panel in (A). The horizontal line indicates 10% duplex formation which defines the upper and lower bounds as previously described. (C) The lower bound, upper bound, Hfq robustness and maximum percentage duplex for each of the four kinetic scenarios described in (A). The color and style of each curve indicates the corresponding topology shown in (A).

**Figure 9 pcbi-1002138-g009:**
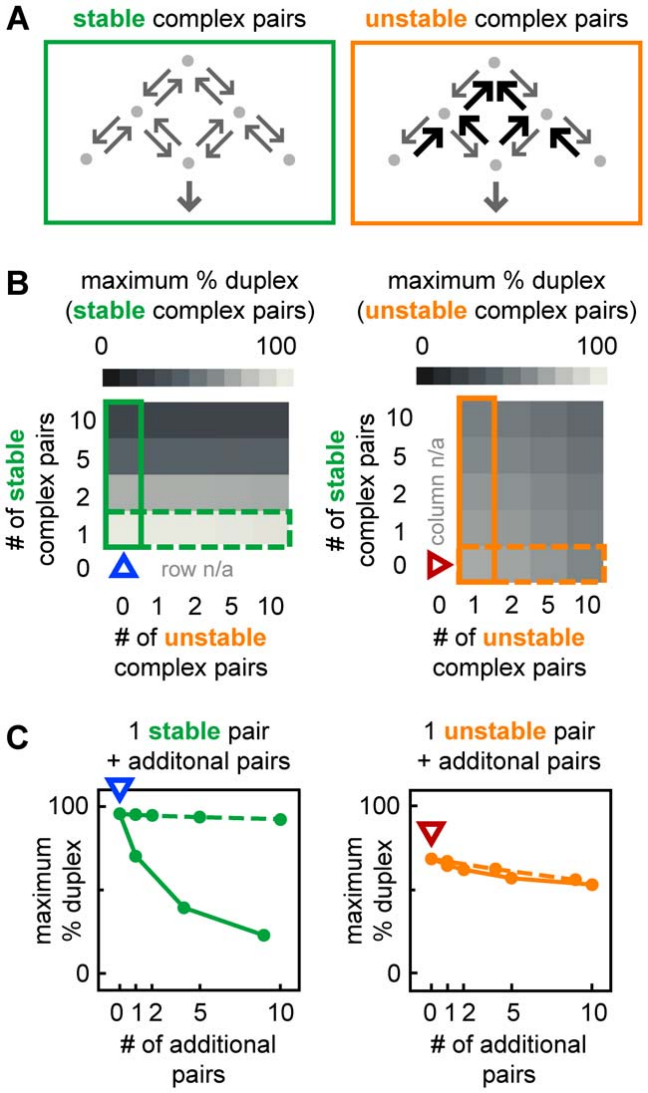
Cognate sRNA-target mRNA pairs with distinct dissociation kinetics perform differently in isolation and in networks. (A) Topologies for cognate pairs which form stable Hfq complexes due to low RNA dissociation rate constants or unstable Hfq complexes due to high RNA dissociation rate constants. (B) Maximum percentage duplex production in networks with different numbers of cognate sRNA-target mRNA pairs that form unstable (horizontal axis) and stable Hfq (vertical axis) complexes. The left and right panels show the maximum duplex formation in cognate pairs that form stable and unstable complexes respectively. The vertical boxed regions (solid green and orange) and the horizontal boxed regions (dash green and orange) indicate the data shown in (C). Blue and red arrowheads indicate stable and unstable cognate pairs acting in isolation. (C) Duplex formation for a single stable pair (left) and a single unstable pair (right) in a network with an increasing number of unstable pairs (dot line) or stable pairs (solid line).

**Figure 10:**
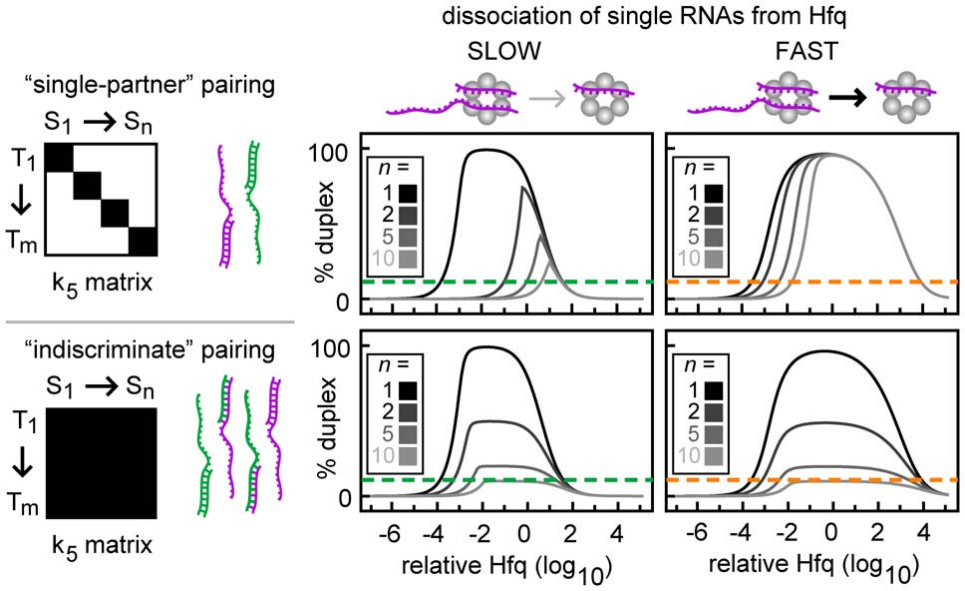
Indiscriminate duplex formation can increase Hfq robustness at the cost of maximum duplex yield. Percentage duplex formed with specific or indiscriminant duplex formation (upper and lower panels) and slow or fast RNA dissociation (left and right panels). For specific duplex formation, each sRNA and target mRNA has only one cognate partner (*i.e.* k_5(i,j)_ = 0 time^−1^ for *i*≠*j* and k_5(i,j)_ = 10^3^ time^−1^ for *i* = *j*) (illustrated in the upper left panel). We define indiscriminate duplex formation as when all sRNAs and target mRNAs can form duplexes with any target mRNA and sRNA respectively (*i.e.* k_5(i,j)_ = 10^3^ time^−1^ for all values of *i* and *j*) (illustrated in the lower left panel). The rate constants for RNA dissociation (k_−1_ = k_−2_ = k_−3_ = k_−4_ = k*_−3_ = k*_−4_ = y_9_) were equal to 10^0^ or 10^4^ time^−1^ for the slow and fast categories respectively. *n* is the number of types of pairs sRNAs and target mRNAs in the network.

#### Non-cognate ternary complexes decrease the efficiency and robustness of duplex formation

The effect of non-cognate ternary complexes on duplex formation was simulated in a system with *n* sRNA-target mRNA pairs, where each sRNA and target mRNA has only one partner (*i.e.* middle right panel of **Figure 5**). In this system there are potentially *n^2^-n* different types of non-cognate ternary complexes that can form (**Figure 6A**). Therefore with five or more sRNA-target mRNA pairs there are more possible types of non-cognate ternary complexes than singly–bound Hfq complexes (*2n*) and cognate ternary complexes (*n*) combined. We demonstrated that when sRNAs and target mRNAs bind independently and with equal affinity for Hfq (**Figure 6B**), duplex formation can be so impaired in this system by the formation of non-cognate ternary complexes that there is no Hfq concentration at which duplexes form efficiently (lower right, **Figure 6C**). Therefore in a network where non-cognate ternary complexes can form, there need to be mechanisms that allow duplexes to form efficiently.

#### Decreasing non-cognate ternary complexes by cooperativity increases the efficiency and robustness of duplex formation

We demonstrate that cooperativity is a mechanism that can increase the proportion of cognate ternary complexes and decrease the proportion of non-cognate ternary complexes. Cooperativity can achieve this by “cognate selection” and “non-cognate exclusion” (**Figure 7A**). Cognate selection requires a stabilizing interaction between cognate sRNA-target mRNA pairs in the ternary complex while non-cognate exclusion requires a destabilizing interaction between non-cognate sRNAs and target mRNAs in the ternary complex.

Cognate selection can occur when Hfq-bound sRNAs or target mRNAs assist the binding of their cognate partner (*i.e.* increasing the RNA binding rate constants k_3_ and k_4_) or when sRNA-target mRNA pairs in cognate ternary complexes stabilize their partners binding (*i.e.* reducing the RNA dissociation rate constants k_−3_ and k_−4_) (**Figures 6B** **,** **7A**). We created a parameter, y_7_, which measures the affinity of sRNAs and target mRNAs for cognate ternary complexes relative to singly-bound complexes [y_7_≡((k_1_⋅k_2_⋅k_−3_⋅k_−4_)/(k_−1_⋅k_−2_⋅k_3_⋅k_4_))^1/4^ which is unitless; y_7_<1 for cognate selection].

Non-cognate exclusion can occur when Hfq-bound sRNAs or target mRNAs occlude the binding sites for non-cognate partners (*i.e.* decreasing the binding rate constants k*_3_ and k*_4_) or when sRNAs and target mRNAs in non-cognate ternary complexes destabilize one another's binding (*i.e.* increasing the dissociation rate constants k*_−3_ and k*_−4_) (**Figures 6B** **,** **7A**). We created a parameter, y_8_, to specify the affinity of sRNAs and target mRNAs for the non-cognate ternary complexes relative to singly-bound complexes [y_8_≡((k_1_⋅k_2_⋅k*_−3_⋅k*_−4_)/(k_−1_⋅k_−2_⋅k*_3_⋅k*_4_))^1/4^ which is unitless; y_8_>1 for non-cognate exclusion].

In the absence of any cooperativity (*i.e.* with independent RNA binding and unbinding), increasing the number of sRNA-target mRNA pairs in the network reduces the maximum percentage duplex achievable and increases the minimum Hfq concentration required for 10% duplex formation (upper left panel in **Figure 7B, C**). The same network with cognate selection or non-cognate exclusion has a greater maximum percentage duplex formation and a lower minimum Hfq concentration for 10% duplex formation (**Figure 7B, C**). Cognate selection combined with non-cognate exclusion reduces Hfq sequestration and improves duplex formation more than either mechanism separately (**Figure 7B, C**). These results show that reducing the sequestration of sRNAs, target mRNAs and Hfq in non-cognate ternary complexes by these forms of cooperativity enables more pairs to signal in parallel over a wider range of Hfq concentrations.

#### Increasing RNA dissociation increases the efficiency and robustness of duplex formation in networks

In this section, rapid RNA dissociation is shown to be an important mechanism for decreasing sequestration in non-cognate ternary complexes (**Figure 8**). The dissociation rate constants determine how stably sRNAs and target mRNAs are bound in non-cognate ternary complexes (and also in cognate ternary complexes). High dissociation kinetics prevent sRNAs and target mRNAs being sequestered in singly-bound Hfq complexes as well as ternary complexes, which allows them to bind Hfq multiple times before they degrade. As a result, there is an increased probability that the sRNAs and target mRNAs will form cognate ternary complexes and this will lead to increased cognate duplex production [if the total magnitude of the association rate constants (y_2_) and duplex annealing and release (k_5_) are sufficiently high].

We created a parameter, y_9_, which measures the total magnitude of RNA dissociation from Hfq complexes [y_9_≡(k_−1_⋅k_−2_⋅k_−3_⋅k*_−3_⋅k_−4_⋅k*_−4_)^1/6^, units of time^−1^] (Note: k*_−3_ and k*_−4_ are defined above). We found that increasing RNA dissociation (y_9_) did indeed decrease the minimum amount of Hfq required for 10% duplex formation (decreased lower bound in **Figure 8B, C**). As before, the impact of duplex annealing and release (k_5_) was examined in combination with RNA dissociation (y_9_) as these reactions compete for cognate ternary complexes. We found that increasing duplex annealing and release together with increased RNA dissociation further decreased the amount of Hfq required for duplex formation. However, increasing duplex annealing and release (k_5_) in the absence of sufficient RNA dissociation had limited effect (**Figure 8B, C**) because the prevalence of the cognate ternary complex is small.

#### Cognate sRNA-target mRNA pairs with distinct dissociation kinetics perform differently in isolation and in networks

We next examined networks with a mix of “stable” cognate pairs that stably bind Hfq due to slow RNA dissociation or “unstable” cognate pairs that unstably bind Hfq due to rapid RNA dissociation (**Figure 9A**). These simulations showed that duplex formation for stable pairs is sensitive to the number of other stable pairs in the network but relatively insensitive to the number of unstable pairs in the network (left panels, **Figure 9B, C**). In contrast, duplex formation for unstable pairs is relatively insensitive to both the number of stable and unstable pairs in the network (right panels, **Figure 9B, C**).

Together the results reveal that in terms of duplex formation, a network is more scalable if it is primarily composed of unstable cognate pairs. The sRNAs and target mRNAs in unstable pairs permit scalability because they do not get sequestered in non-cognate ternary complexes due to rapid RNA dissociation. Whereas cognate pairs that form stable Hfq complexes and perform well in isolation, may perform relatively poorly in a network. This result shows that an assessment of the properties of cognate pairs needs to take into account the other sRNAs and target mRNAs that are acting at the same time; this has important implications for future studies as we discuss below.

#### Indiscriminate duplex formation can increase Hfq robustness at the cost of decreased maximum duplex yield

Until this point, we have only simulated networks where sRNAs and target mRNAs have one cognate partner. Here, we directly compare a network where sRNAs and target mRNAs have one cognate partner (upper panels, **Figure 10**) to a network where all ternary complexes can form duplexes (lower panels, **Figure 10**). In other words, in the latter network all sRNAs and target mRNAs can form a duplex with any other RNA (*i.e.* k_5(i,j)_>0, for all values of *i* and *j*); this represents ubiquitous cross-talk or non-specific interactions. Of course in a wild-type network not all sRNAs and target mRNAs will indiscriminately form duplexes with each other. However, this scenario will most clearly shed light on how the formation of multiple types of duplexes by each type of sRNA and target mRNA, which does occur to some extent in wild-type sRNA networks, affects the efficiency and robustness of duplex formation.

We found that at low rates of RNA dissociation, the indiscriminate formation of duplexes prevents Hfq sequestration because there are no non-cognate ternary complexes and therefore duplexes are released from all ternary complexes (upper and lower left panels, **Figure 10**). This increases Hfq recycling which decreases the lower bound resulting in increased Hfq robustness. However, the increased Hfq robustness comes at the cost of a large reduction in the maximum yield for any given duplex. The amount of duplex decreases because sRNAs and target mRNAs are being incorporated into a wider variety of duplexes and therefore there is less cognate sRNA and target mRNA for any particular duplex. It should be noted that indiscriminate sRNA-target mRNA pairing has little impact on Hfq robustness when RNA dissociation from Hfq complexes is high. This is because under these conditions Hfq recycling is already high (upper and lower right panels, **Figure 10**) and therefore the sequestration of Hfq and RNAs in non-cognate ternary complexes is low.

#### Imbalances in sRNA and target mRNA production can globally alter duplex formation

**Figure 11:**
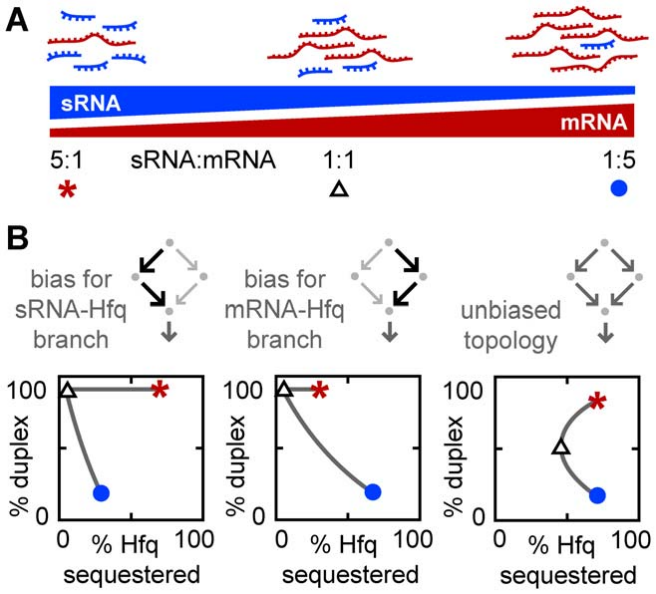
Sequestration of Hfq by imbalanced RNA expression is minimized by a compulsory RNA binding order. (A) The relative concentration of sRNA to target mRNA within a cognate sRNA-target mRNA pair is varied from 5∶1 to 1∶5 while the combined concentration of sRNA and target mRNA is kept constant. sRNA/target mRNA ratios of 5∶1, 1∶1 and 1∶5 are indicated by a red asterisk, black triangle and blue circle respectively. (B) Percentage duplex versus percentage Hfq sequestered for cognate sRNA-target mRNA pairs with imbalanced ratios. The shape of the curve depends on whether the kinetics of the sRNA-target mRNA pair is biased towards the sRNA-Hfq branch, the target mRNA-Hfq branch or neither. The bias was altered by changing the values for y_3_ while keeping y_1_, y_2_ and y_4_ constant (y_3_ = 10^−8^, 10^8^ and 10^0^ in the left, middle and right panels respectively).

In the previous simulations, the production and degradation rates were equal for both members of each cognate sRNA-target mRNA pair. We now investigate how unequal production rates for sRNAs and target mRNAs impacts duplex formation in different scenarios (**Figure 11** **–** [Fig pcbi-1002138-g012] **13**).

**Figure 12 pcbi-1002138-g012:**
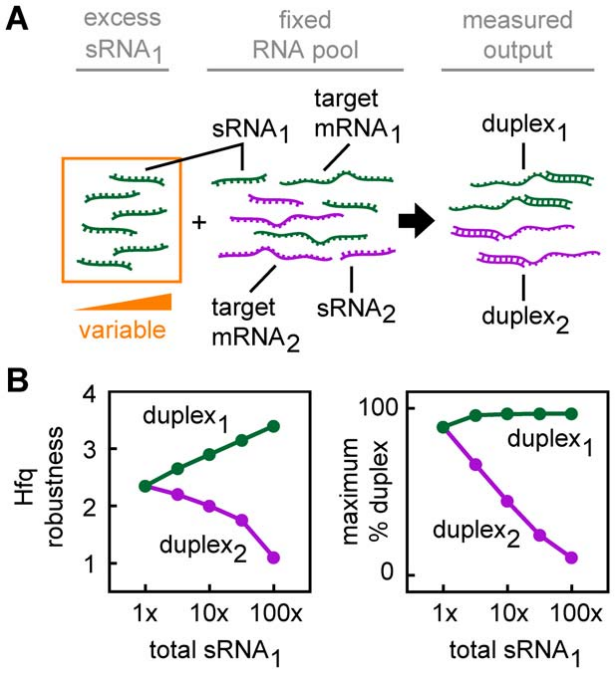
Imbalances in sRNA and target mRNA production can globally alter duplex formation. (A) A network with two cognate sRNA-target mRNA pairs where each sRNA and target mRNA can only bind one partner. Initially the concentrations of sRNA_1_, sRNA_2_, target mRNA_1_ and target mRNA_2_ are equal. Additional sRNA_1_ is then added to the system by increasing the production of sRNA_1_. (B) Hfq robustness and the maximum percentage duplex for the sRNA_1_-target mRNA_1_ pair (green) and the sRNA_2_-target mRNA_2_ pair (purple) as a function of the total concentration of sRNA_1_.The total sRNA_1_ concentration is measured relative to target mRNA_1_.

**Figure 13:**
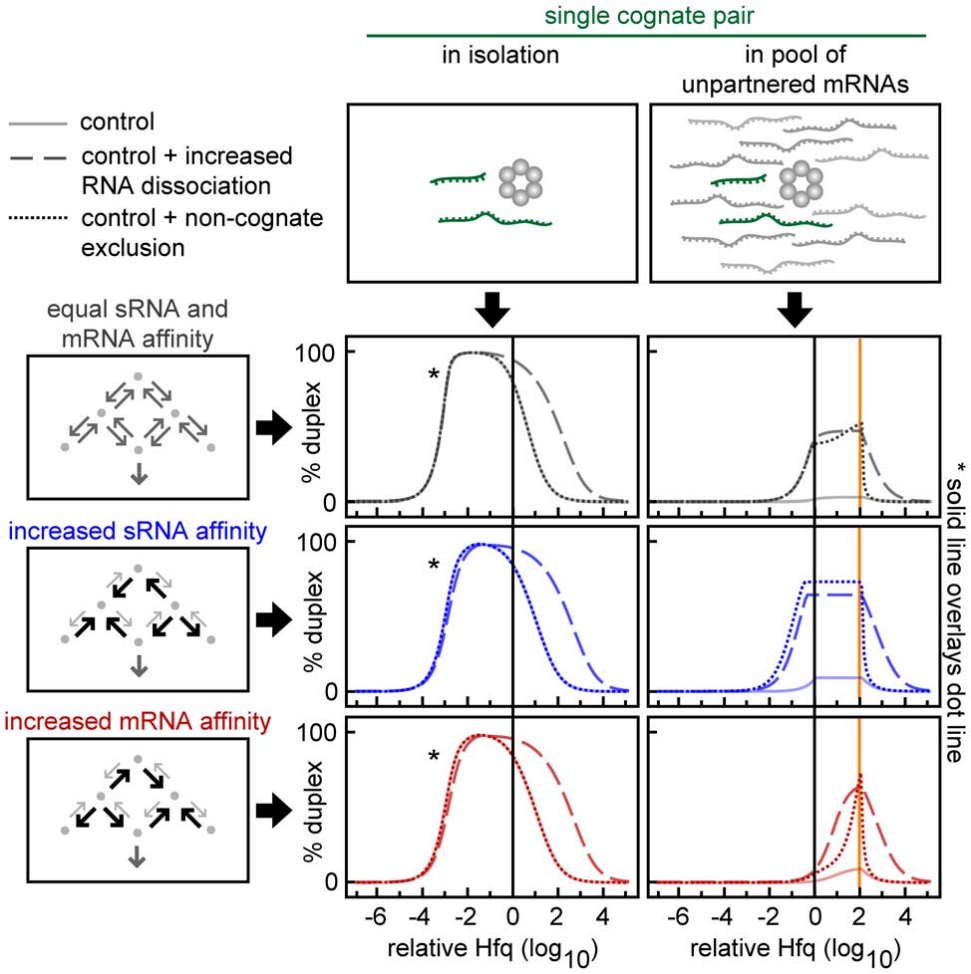
Duplex formation and Hfq robustness in the presence of excess, unpartnered target mRNAs. Duplex formation for a cognate sRNA-target mRNA pair in the absence (left panels) and presence (right panels) of competing non-cognate mRNAs. The sRNA only forms duplexes with its cognate target mRNA. Each plot shows a control topology with moderate RNA dissociation and no cooperativity (solid line), the control topology with increased RNA dissociation for all reaction steps (dash line) and the control topology with non-cognate exclusion which was created by selectively increasing RNA dissociation from non-cognate duplexes (dot line). The affinity of the cognate sRNA and target mRNA for Hfq was varied by increasing and decreasing two groups of rate constants (k_1_, k_4_, k*_4_ and k_2_, k_3_ and k*_3_) so that the ratio ((k_2_·k_3_·k*_3_)/(k_1_·k_4_·k*_4_))^1/3^ had values of 10^0^, 10^−2^ and 10^2^ (equal affinity, high sRNA affinity and high target mRNA affinity respectively). In these simulations, total RNA dissociation which was measured by y_9_ had values of 10^0^, 10^4^ and 10^1^ (top, middle and bottom panels respectively). Non-cognate exclusion which was measured by y_8_ had values of 10^0^, 10^0^ and 10^1^ (top, middle and bottom panels respectively). The vertical black line indicates where the Hfq concentration equals the total target mRNA concentration in the cognate pair. The vertical yellow line indicates where the Hfq concentration equals the concentration of all competing, non-cognate mRNAs.

We first show that for a single cognate sRNA-target mRNA pair there will be less Hfq sequestration if the member of the pair that is in excess does not efficiently bind free Hfq (**Figure 11**). If the concentration of the target mRNA exceeds its sRNA partner (blue circles in **Figure 11**) then sequestration will be lower if only the sRNA binds free Hfq (*i.e.* a sRNA-Hfq branch bias). Conversely, if the concentration of sRNA exceeds its target mRNA partner (red asterisks in **Figure 11**), sequestration will be less if only the target mRNA binds free Hfq (*i.e.* a target mRNA-Hfq branch bias).

We next examined a simple network composed of two cognate sRNA-target mRNA pairs (sRNA_1_-target mRNA_1_ and sRNA_2_-target mRNA_2_) where each RNA can only form duplexes with one partner. The production of one sRNA (sRNA_1_) was varied and the production of the other RNAs was kept constant and equal (*i.e.* the production of sRNA_1_ ≥ production of sRNA_2_, target mRNA_1_ and target mRNA_2_) (**Figure 12A**). All sRNAs and target mRNAs had the same degradation rate, independent binding to Hfq and identical RNA dissociation kinetics. In this scenario, increasing sRNA_1_ production increased Hfq robustness for the sRNA_1_-target mRNA_1_ pair because the additional sRNA drives the concentration of the sRNA_1_-Hfq complex higher which increases the formation of the sRNA_1_-Hfq-target mRNA_1_ ternary complex and duplex_1_ (**Figure 12B**). However, the excess sRNA_1_-Hfq also leads to increased formation of non-cognate ternary complexes which diminishes duplex formation and robustness for the other sRNA-target mRNA pair (duplex_2_ in **Figure 12B**). That is, imbalances in sRNA-target mRNA concentrations can increase Hfq robustness for one pair at the cost of reducing duplex formation and Hfq robustness for other pairs.

We then investigated a common scenario where most target mRNAs are transcribed without their partners and a single sRNA is activated in response to a specific stressor [38], [39]. In this case, a single cognate sRNA-target mRNA pair with balanced production needs to signal in a pool of unpartnered target mRNAs (**Figure 13**). The large pool of unpartnered target mRNAs (100-fold the concentration of the cognate target mRNA in this simulation) increases the formation of non-cognate ternary complexes and therefore decreases Hfq recycling. The decreased Hfq recycling results in an increased upper bound and decreased duplex formation (compare right and left panels, **Figure 13**).

As with the other scenarios described above, rapid RNA dissociation and cooperativity (only non-cognate exclusion is shown) minimize non-cognate ternary complexes resulting in increased Hfq robustness and duplex formation (upper panels, **Figure 13**). Hfq robustness is also improved by increasing the affinity of the sRNA for Hfq (middle panels, **Figure 13**) because this increases the probability that the limited numbers of sRNA that are transcribed in response to a specific stressor are bound to Hfq. Therefore when the cognate target mRNA binds to Hfq there is a higher probability that the cognate ternary complex will form. Because there is a large pool of target mRNAs, increasing the affinity of the target mRNAs (cognate and unpartnered) for Hfq reduces the fraction of target mRNAs that have the chance to bind Hfq in a given period. This lowers the probability that the cognate target mRNA will be bound to one of the limited number of Hfq hexamers and diminishes the robustness of the cognate pair (lower panels, **Figure 13**). Of course, selectively increasing only the affinity of the cognate target mRNA for Hfq would increase Hfq robustness and duplex formation for the cognate pair, but this would only work if the same sRNA was always induced in response to stress; this is clearly not a realistic scenario.

In summary, imbalances in sRNA and target mRNA production alter Hfq robustness and maximum duplex formation and some of these effects are only apparent in the context of a network. We demonstrated that an excess of a particular sRNA can increase the efficiency of forming its duplexes but this occurs at the cost of decreased duplex formation and Hfq robustness for other sRNAs. Strategies that reduce non-cognate ternary complex formation such as bias for the sRNA-Hfq or target mRNA-Hfq branch, cooperativity, rapid RNA dissociation, and differences in the affinity of sRNAs and target mRNAs for Hfq can help compensate for imbalanced and unpartnered RNAs.

## Discussion

In this study we investigated a fundamental question; how is Hfq able to efficiently and robustly mediate duplex formation in the internal environment of the cell with many sRNAs and target mRNAs competing for Hfq? The difficulty of this task is exacerbated by the fact that the Hfq concentration varies with environmental cues [33], [34], [35] as does the number, types and concentrations of the sRNAs and target mRNAs [39], [40], [41]. To address this question we modeled the kinetics of the interactions between Hfq, sRNAs and target mRNAs which are the foundation of sRNA networks.

The model used in this study is very general and the basic qualitative findings are likely to apply to other realistic binding scenarios. For example, if sRNAs and target mRNAs compete for the same sites on Hfq rather than having separate binding sites as we have modeled, then this would simply increase the number of possible non-cognate ternary complexes. In this case there would be 2*n* ^2^–*n* non-cognate ternary complexes instead of *n* ^2^–*n* (where *n* is the number of sRNA-target mRNA pairs) in networks with single-partner pairing. While this reduces Hfq availability, it does not alter the fundamental kinetics of the system except that sRNA-target mRNA pairs acting in isolation would also require a mechanism to prevent non-cognate ternary complexes from forming [*i.e.* (sRNA)_2_-Hfq and (target mRNA)_2_-Hfq]. Similarly, the binding of Hfq to DNA [15], [16] and polyadenylated RNAs [13], [17] will decrease Hfq availability but it will not alter the basic kinetics of duplex formation.

The model was used to systematically explore the possible kinetic mechanisms for increasing the efficiency and robustness of sRNAs. We found two non-mutually exclusive mechanisms that can help achieve these goals: 1. heterotropic cooperativity; and 2. rapid RNA dissociation from Hfq complexes. Both mechanisms can increase duplex formation and/or robustness for single cognate sRNA-target mRNA pairs acting in isolation, in networks with many competing sRNAs and target mRNAs, and when there are imbalances in sRNA and target mRNA concentrations.

Cooperativity can enhance duplex formation by increasing the proportion of bound Hfq in cognate ternary complexes. This can occur by increasing the formation and/or stability of cognate ternary complexes (*i.e.* cognate selection) or by decreasing the formation and/or stability of non-cognate ternary complexes (*i.e.* non-cognate exclusion) and singly-bound Hfq complexes. The “active” cycling model that was recently proposed [23], where the binding of one RNA to Hfq increases the dissociation of another RNA bound to Hfq, is consistent with what we have termed non-cognate exclusion. There is *in vitro* experimental evidence which is consistent with our definition for cooperativity. Kinetics measurements with synthetic RNA sequences have shown unequal formation of duplexes via the sRNA-Hfq and target mRNA-Hfq branches; this indicates that RNA binding to Hfq is affected by the presence of other RNAs bound to Hfq [28]. Furthermore, sRNAs and target mRNAs dissociate from non-cognate ternary complexes at greater rates than from singly-bound Hfq complexes which is consistent with non-cognate exclusion [23], [42]. There is currently no clear evidence for or against cognate selection.

In contrast to cooperativity, high RNA dissociation rates do not alter the relative proportion of Hfq complexes but instead increase the cycling of sRNAs and target mRNAs among cognate and non-cognate ternary complexes and singly-bound Hfq complexes. The increased cycling improves the likelihood that sRNAs and target mRNA are incorporated into a cognate ternary complex before they degrade. Rapid RNA dissociation has been observed *in vitro* for non-cognate ternary complexes [23] and for cognate ternary complexes [28]. The evidence for rapid RNA dissociation from singly-bound Hfq complexes is less clear; singly-bound Hfq complexes have reported half-lives (which is a measure of the rate of RNA dissociation) in the absence of competition which varies from approximately 165 minutes [23] to 0.03 s [28] to between 2.1×10^−5^ and 4.1×10^−5^ s [42].

How sRNAs and target mRNAs interact while bound to Hfq is unclear. It may involve allosteric changes in the Hfq hexamer, electrostatic interactions or direct interactions between the sRNAs and/or target mRNAs bound to Hfq. Whatever the mechanism, *in vivo* experiments suggest that the 5′ sequences of sRNAs and target mRNAs are in at least some cases sufficient to ensure specific interactions between the sRNAs and the target mRNAs [43]. Furthermore, a comparative analysis of sRNAs across different genomes has shown that the binding sequences, irrespective of their location, are the only regions that are consistently conserved [44].

We demonstrated that duplex formation not only depends on the kinetics of the sRNA-target mRNA pair of interest but it also depends on the kinetics and abundance of competing RNAs. This was highlighted by showing that in isolation sRNA-target mRNA pairs that form stable Hfq complexes can perform better than pairs that form unstable Hfq complexes, whereas in a sRNA network the reverse can occur. Further illustrating the importance of the environment on duplex formation, we showed that excess production of a sRNA can be used to selectively enhance the formation of one type of duplex at the cost of decreased duplex formation for other sRNA-target mRNA pairs. Similarly, we demonstrated that a pool of unpartnered target mRNAs can alter duplex formation for a cognate pair.

It is clear that more *in vitro* and *in vivo* experimental data is needed to understand duplex formation in biologically realistic scenarios where there is competition for Hfq. In particular it is important to ascertain which of the two mechanisms for reducing sequestration (cooperativity or high RNA dissociation) is more important and whether it varies among sRNAs. This could be determined by isolating singly-bound Hfq complexes, cognate ternary complexes and non-cognate ternary complexes and measuring their association and dissociation rate constants via electrophoretic mobility shift assays, surface plasmon resonance and filter binding assays [23], [42]. Furthermore, because duplex formation is dependent on the number, types and concentrations of the other RNAs that are competing for Hfq, *in vivo* measurement of these factors under specific physiological conditions is desirable to obtain an accurate quantification of sRNA activity. Genome wide identification and quantification of sRNAs and target mRNAs that compete for Hfq could be obtained by deep sequencing expression analysis [45], [46]. Of course there is also a need to determine the availability of Hfq under the same conditions. Direct measurement of Hfq concentration [34], [47] may not be helpful because Hfq has the capacity to bind DNA and other proteins and therefore the available fraction of Hfq would be difficult to ascertain. It may be more practical to directly measure Hfq availability and competition using a reporter system [22].

It is important to acknowledge that even with mechanisms such as cooperativity and rapid RNA dissociation acting to optimize Hfq function, the sRNA network may not signal effectively under some conditions due to Hfq competition. Recently, it was shown *in vivo* that over-expressing a single sRNA or target mRNA can be sufficient to generally disrupt sRNA signaling due to Hfq competition [22]. The inability of Hfq to mediate sRNA signaling under only certain conditions could be a desirable feature when individual signals (*e.g.* iron deficiency, oxidative stress, nutrient limitation) need to be over-ridden or controlled centrally under specific conditions such as pathogenesis or stress. Cells could globally turn off or tune the activity of large subsets of sRNAs by varying the production of Hfq or the transcription of RNA competitors.

In conclusion, there are simple kinetic mechanisms that can increase the efficiency and robustness of Hfq activity to enable it to mediate multiple sRNA signals in parallel. These mechanisms are cooperativity and/or rapid RNA dissociation which minimize the sequestration of sRNAs, target mRNAs and Hfq. Determining the role of these mechanisms *in vivo* will require further characterization of the composition and kinetics of sRNA networks in cells under physiologically relevant conditions. The general model we have presented is a valuable framework to guide, analyze and interpret these future experiments.

## Model

In our model [H], [S_i_] and [T_j_] represent the concentrations of unbound Hfq hexamer, the *i*th unbound sRNA and the *j*th unbound target mRNA respectively. [HS_i_] and [HT_j_] denote singly-bound Hfq hexamers with the *i*th sRNA and the *j*th target mRNA respectively. [HS_i_T_j_] denotes a ternary complex where Hfq is bound to the *i*th sRNA and the *j*th target mRNA. [D_i_,_j_] denotes the sRNA-target mRNA duplex formed by the combination of the *i*th sRNA and the *j*th target mRNA. In this model, Hfq cannot bind more than one sRNA or target mRNA.

The number of differential equations needed to describe a network depends on number of types of sRNA and target mRNA species. In a network with *n* types of sRNA species (*i.e. i* = 1 to *n*) and *m* types of target mRNA species (*i.e. j* = 1 to *m*) there will be: 1. a single equation that describes the dynamics of free Hfq; 2. *n* equations that describe the dynamics of free sRNAs; 3. *m* equations that describe the dynamics of free mRNAs; 4. *n* equations that describe the dynamics of sRNA-Hfq complexes; 5. *m* equations that describe the dynamics of target mRNA-Hfq complexes; 6. *n*⋅*m* equations that describe the dynamics of ternary Hfq complexes; and 7. *n*⋅*m* equations that describe the dynamics of sRNA-target mRNA duplexes.
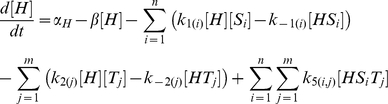


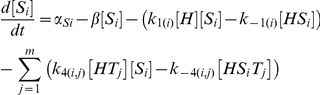


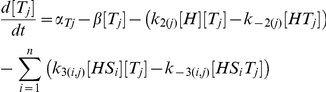


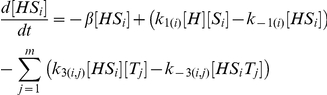


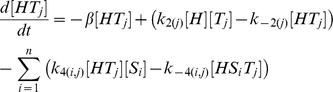


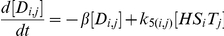


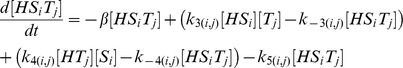



The parameters *α*_H_, *α*_Si_ and *α*_Tj_ indicate the production rates of Hfq, the *i*th sRNA and the *j*th target mRNA respectively. k_1(i)_, k_−1(i)_, k_2(j)_ and k_−2(j)_ describe the formation and dissociation of singly-bound Hfq complexes and k_3(i,j)_, k_−3(i,j)_, k_4(i,j)_, k_−4(i,j)_ describe the formation and dissociation of ternary complexes. The *i* subscript in these rate constants denotes which of the *n* sRNAs is binding or unbinding and the *j* subscript denotes which of the *m* target mRNAs is binding or unbinding. k_5(i,j)_ specifies the formation and release of a sRNA-target mRNA duplex from a Hfq ternary complex and is zero for non-cognate sRNA-target mRNA pairs (*i.e.* when *i*≠*j*) unless otherwise stated. As explained above, the rate constant for degradation and dilution (*β*) was the same for all species to keep the model as simple as possible; this simplification does not alter the basic qualitative results (**Figure S1**).

We did not include the possibility of Hfq dodecamers and the exchange of RNAs between Hfq hexamers (which must also form a Hfq dodecameric complex) in the basic model [48], [49]. This would have required the creation of additional reaction paths and therefore increased complexity for the basic model. Furthermore, Hfq dodecamers have not been consistently observed *in vivo* and a recent study has shown that single Hfq hexamers are sufficient for duplex formation [50]. Of course the latter does not exclude a role for Hfq dodecamers in duplex formation but current evidence suggests it is not essential.

The parameter values for the simulations are provided in the **Supporting Information Table S1**. Many of the kinetic parameters for duplex formation have not been quantified or the reported values vary widely (see **Discussion**). For this reason, the values for the kinetic parameters were given arbitrary values and these were varied over several orders of magnitude to ensure that biologically relevant regimes are likely to fall within the ranges selected.

All the data presented in this study is generated from the steady state solutions to the differential equations described above by allowing numerically generated time courses to run to convergence using the NDSolve subroutine of Mathematica 6 (Wolfram Research, Champaign, IL). Simulations were run for at least 100 times the RNA lifetime and convergence was confirmed by ensuring that the solutions at 90% and 100% of the simulation differed by no more 1×10^−10^.

## Supporting Information

Figure S1 **Varying sRNA degradation minimally affects duplex formation.** The effect of the free sRNA degradation rate constant on duplex formation was examined in a system with independent sRNA and target mRNA binding (left panels), positive cooperative association (middle panels) and rapid RNA dissociation (right panels). For native sRNAs the degradation rate is typically greater than for target mRNAs and Hfq. Therefore we increased free sRNA degradation by 10-fold (compared to the simulations in the main text). The degradation of the target mRNA, Hfq and Hfq complexes were unchanged. The simulations showed that increasing the degradation of free sRNA compared to the target mRNA and Hfq had minimal effect on duplex formation (Note: the simulated difference is greater than typically occurs physiologically). The grey dash line indicates a 1∶1 ratio of [total target mRNA] to [Hfq], where the [total target mRNA]≡[T]+[HT]+[HST]+[D]; [T], [HT], [HST] and [D] are the concentrations of free target mRNA, target mRNA-Hfq complex, cognate ternary complex and duplex respectively.
(TIF)

Table S1 **Kinetic parameters used in the simulations.** Abbreviations used to specify the panels in the figures are: top left (TL), top center (TC), top right (TR), middle left (ML), middle center (MC), middle right (MR), bottom left (BL), bottom center (BC) and bottom right (BR). ‡ indicates sRNA-target mRNA pairs that form “stable” complexes with Hfq and ◊ indicates “unstable” complexes with Hfq (Figure 9U). £ Two sRNA-target mRNA pairs were simulated which have the same kinetic parameters (both panels represent the same set of simulations with varying sRNA_1_ production). ¶ Control topologies (solid curves). ¥ Topologies with increased RNA dissociation relative to the control (dash curves). § Topologies exhibiting non-cognate exclusion (dot curves). Hfq production (α_H_) in all panels is varied from 10^−5^ to 10^7^ concentration·time^−1^ unless otherwise indicated. conc. = concentration.
(DOC)
