# Supplementary figures and images for: Essential Requirements for Robust Signaling in Hfq Dependent Small RNA Networks
Source: PLoS Comput Biol. 2011 Aug 18;7(8):e1002138. doi: 10.1371/journal.pcbi.1002138 (PMC3158044; doi:10.1371/journal.pcbi.1002138)

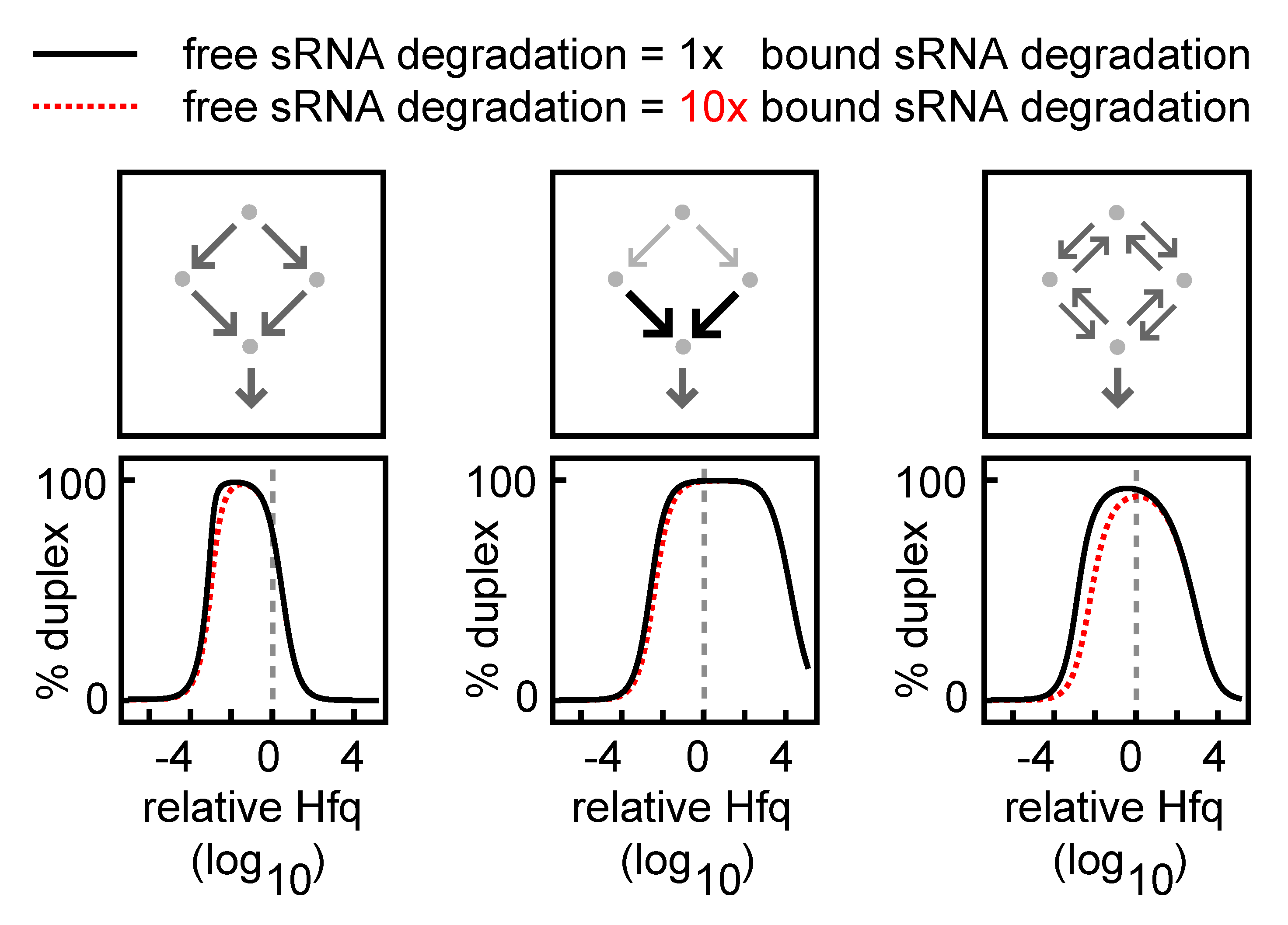

Supplement: Figure S1 — Varying sRNA degradation minimally affects duplex formation. The effect of the free sRNA degradation rate constant on duplex formation was examined in a system with independent sRNA and target mRNA binding (left panels), positive cooperative association (middle panels) and rapid RNA dissociation (right panels). For native sRNAs the degradation rate is typically greater than for target mRNAs and Hfq. Therefore we increased free sRNA degradation by 10-fold (compared to the simulations in the main text). The degradation of the target mRNA, Hfq and Hfq complexes were unchanged. The simulations showed that increasing the degradation of free sRNA compared to the target mRNA and Hfq had minimal effect on duplex formation (Note: the simulated difference is greater than typically occurs physiologically). The grey dash line indicates a 1∶1 ratio of [total target mRNA] to [Hfq], where the [total target mRNA]≡[T]+[HT]+[HST]+[D]; [T], [HT], [HST] and [D] are the concentrations of free target mRNA, target mRNA-Hfq complex, cognate ternary complex and duplex respectively. (TIF) [file pcbi.1002138.s001.tif]
