# Supplementary material for: Essential Requirements for Robust Signaling in Hfq Dependent Small RNA Networks
Source: PLoS Comput Biol. 2011 Aug 18;7(8):e1002138. doi: 10.1371/journal.pcbi.1002138 (PMC3158044; doi:10.1371/journal.pcbi.1002138)
Supplement: Table S1 — Kinetic parameters used in the simulations. Abbreviations used to specify the panels in the figures are: top left (TL), top center (TC), top right (TR), middle left (ML), middle center (MC), middle right (MR), bottom left (BL), bottom center (BC) and bottom right (BR). ‡ indicates sRNA-target mRNA pairs that form “stable” complexes with Hfq and ◊ indicates “unstable” complexes with Hfq (Figure 9U). £ Two sRNA-target mRNA pairs were simulated which have the same kinetic parameters (both panels represent the same set of simulations with varying sRNA1 production). ¶ Control topologies (solid curves). ¥ Topologies with increased RNA dissociation relative to the control (dash curves). § Topologies exhibiting non-cognate exclusion (dot curves). Hfq production (αH) in all panels is varied from 10−5 to 107 concentration·time−1 unless otherwise indicated. conc. = concentration. (DOC) [file pcbi.1002138.s002.doc]

**Supporting Information Table S1. Kinetic parameters used in the simulations.**

| **Fig 2C** | **units** | **TL** | **TC** | **TR** | **ML** | **MC** | **MR** | **BL** | **BC** | **BR** |
| --- | --- | --- | --- | --- | --- | --- | --- | --- | --- | --- |
| k_1_ | conc.^-1^·time^-1^ | 10^6.5^ | 10^4.5^ | 10^2.5^ | 10^4.5^ | 10^2.5^ | 10^0.5^ | 10^2.5^ | 10^0.5^ | 10^-1.5^ |
| k_2_ | conc.^-1^·time^-1^ | 10^2.5^ | 10^4.5^ | 10^6.5^ | 10^0.5^ | 10^2.5^ | 10^4.5^ | 10^-1.5^ | 10^0.5^ | 10^2.5^ |
| k_3_ | conc.^-1^·time^-1^ | 10^2.5^ | 10^4.5^ | 10^6.5^ | 10^0.5^ | 10^2.5^ | 10^4.5^ | 10^-1.5^ | 10^0.5^ | 10^2.5^ |
| k_4_ | conc.^-1^·time^-1^ | 10^6.5^ | 10^4.5^ | 10^2.5^ | 10^4.5^ | 10^2.5^ | 10^0.5^ | 10^2.5^ | 10^0.5^ | 10^-1.5^ |
| k_5_ | time^-1^ | 10^3^ | 10^3^ | 10^3^ | 10^3^ | 10^3^ | 10^3^ | 10^3^ | 10^3^ | 10^3^ |
| k_-1,-2,-3,-4_ | time^-1^ | 0 | 0 | 0 | 0 | 0 | 0 | 0 | 0 | 0 |
| α_S,T_ | conc.·time^-1^ | 10^2^ | 10^2^ | 10^2^ | 10^2^ | 10^2^ | 10^2^ | 10^2^ | 10^2^ | 10^2^ |
| β | time^-1^ | 10^0^ | 10^0^ | 10^0^ | 10^0^ | 10^0^ | 10^0^ | 10^0^ | 10^0^ | 10^0^ |
| **Fig 3C** | **units** | **TL** | **TC** | **TR** | **ML** | **MC** | **MR** | **BL** | **BC** | **BR** |
| k_1_ | conc.^-1^·time^-1^ | 10^6.5^ | 10^4.5^ | 10^2.5^ | 10^4.5^ | 10^2.5^ | 10^0.5^ | 10^2.5^ | 10^-1.5^ | 10^-1.5^ |
| k_2_ | conc.^-1^·time^-1^ | 10^2.5^ | 10^4.5^ | 10^6.5^ | 10^0.5^ | 10^2.5^ | 10^4.5^ | 10^-1.5^ | 10^-1.5^ | 10^2.5^ |
| k_3_ | conc.^-1^·time^-1^ | 10^2.5^ | 10^0.5^ | 10^-1.5^ | 10^4.5^ | 10^2.5^ | 10^0.5^ | 10^6.5^ | 10^6.5^ | 10^2.5^ |
| k_4_ | conc.^-1^·time^-1^ | 10^-1.5^ | 10^0.5^ | 10^2.5^ | 10^0.5^ | 10^2.5^ | 10^4.5^ | 10^2.5^ | 10^6.5^ | 10^6.5^ |
| k_5_ | time^-1^ | 10^3^ | 10^3^ | 10^3^ | 10^3^ | 10^3^ | 10^3^ | 10^3^ | 10^3^ | 10^3^ |
| k_-1,-2,-3,-4_ | time^-1^ | 0 | 0 | 0 | 0 | 0 | 0 | 0 | 0 | 0 |
| α_S,T_ | conc.·time^-1^ | 10^2^ | 10^2^ | 10^2^ | 10^2^ | 10^2^ | 10^2^ | 10^2^ | 10^2^ | 10^2^ |
| β | time^-1^ | 10^0^ | 10^0^ | 10^0^ | 10^0^ | 10^0^ | 10^0^ | 10^0^ | 10^0^ | 10^0^ |
| **Fig 3F** | **units** | **TL** | **TC** | **TR** | **ML** | **MC** | **MR** | **BL** | **BC** | **BR** |
| k_1,2,3,4_ | conc.^-1^·time^-1^ | 10^2.5^ | 10^2.5^ | 10^2.5^ | 10^2.5^ | 10^2.5^ | 10^2.5^ | 10^2.5^ | 10^2.5^ | 10^2.5^ |
| k_5_ | time | 10^0^ | 10^3^ | 10^6^ | 10^0^ | 10^3^ | 10^6^ | 10^0^ | 10^3^ | 10^6^ |
| k_-1,-2,-3,-4_ | time | 10^8^ | 10^8^ | 10^8^ | 10^4^ | 10^4^ | 10^4^ | 10^0^ | 10^0^ | 10^0^ |
| α_S,T_ | conc.·time^-1^ | 10^2^ | 10^2^ | 10^2^ | 10^2^ | 10^2^ | 10^2^ | 10^2^ | 10^2^ | 10^2^ |
| β | time^-1^ | 10^0^ | 10^0^ | 10^0^ | 10^0^ | 10^0^ | 10^0^ | 10^0^ | 10^0^ | 10^0^ |
| **Fig 4A** | **units** | **TL** | **TC** | **TR** | **ML** | **MC** | **MR** | **BL** | **BC** | **BR** |
| k_1,2_ | conc.^-1^·time^-1^ | 10^2.5^ |  | 10^2.5^ |  |  |  | 10^2.5^ |  | 10^0.5^ |
| k_3,4_ | conc.^-1^·time^-1^ | 10^2.5^ |  | 10^2.5^ |  |  |  | 10^2.5^ |  | 10^4.5^ |
| k_5_ | time^-1^ | 10^3^ |  | 10^3^ |  |  |  | 10^3^ |  | 10^3^ |
| k_-1,-2,-3,-4_ | time^-1^ | 0 |  | 0 |  |  |  | 10^4^ |  | 10^4^ |
| α_S,T_ | conc.·time^-1^ | 10^2^ |  | 10^2^ |  |  |  | 10^2^ |  | 10^2^ |
| β | time^-1^ | 10^0^ |  | 10^0^ |  |  |  | 10^0^ |  | 10^0^ |
| **Fig 4B** | **Units** | **TL** | **TC** | **TR** | **ML** | **MC** | **MR** | **BL** | **BC** | **BR** |
| k_1,2_ | conc.^-1^·time^-1^ | 10^2.5^ |  | 10^0.5^ |  |  |  | 10^2.5^ |  | 10^0.5^ |
| k_3,4_ | conc.^-1^·time^-1^ | 10^2.5^ |  | 10^4.5^ |  |  |  | 10^2.5^ |  | 10^4.5^ |
| k_5_ | time^-1^ | 10^3^ |  | 10^3^ |  |  |  | 10^3^ |  | 10^3^ |
| k_-1,-2_ | time^-1^ | 10^4^ |  | 10^4^ |  |  |  | 10^6^ |  | 10^6^ |
| k_-3,-4_ | time^-1^ | 10^4^ |  | 10^4^ |  |  |  | 10^2^ |  | 10^2^ |
| α_S,T_ | conc.·time^-1^ | 10^2^ |  | 10^2^ |  |  |  | 10^2^ |  | 10^2^ |
| β | time^-1^ | 10^0^ |  | 10^0^ |  |  |  | 10^0^ |  | 10^0^ |
| **Fig 6C** | **Units** | **TL** | **TC** | **TR** | **ML** | **MC** | **MR** | **BL** | **BC** | **BR** |
| k_1,2,3,4_ | conc.^-1^·time^-1^ | 10^2.5^ |  | 10^2.5^ |  |  |  | 10^2.5^ |  | 10^2.5^ |
| k_5_ | time^-1^ | 10^3^ |  | 10^3^ |  |  |  | 10^3^ |  | 10^3^ |
| k_-1,-2,-3,-4_ | time^-1^ | 10^0^ |  | 10^0^ |  |  |  | 10^0^ |  | 10^0^ |
| α_S,T_ | conc.·time^-1^ | 10^2^ |  | 10^2^ |  |  |  | 10^2^ |  | 10^2^ |
| β | time^-1^ | 10^0^ |  | 10^0^ |  |  |  | 10^0^ |  | 10^0^ |

**Supporting Information Table S1. (continued)**

| **Fig 7B,C** | **units** | **TL** | **TC** | **TR** | **ML** | **MC** | **MR** | **BL** | **BC** | **BR** |
| --- | --- | --- | --- | --- | --- | --- | --- | --- | --- | --- |
| k_1,2_ | conc.^-1^·time^-1^ | 10^2.5^ |  | 10^2.5^ |  |  |  | 10^2.5^ |  | 10^2.5^ |
| k_3,4_ | conc.^-1^·time^-1^ | 10^2.5^ |  | 10^3.5^ |  |  |  | 10^2.5^ |  | 10^3.5^ |
| k*_3,4_ | conc.^-1^·time^-1^ | 10^2.5^ |  | 10^2.5^ |  |  |  | 10^1.5^ |  | 10^1.5^ |
| k_5_ | time^-1^ | 10^3^ |  | 10^3^ |  |  |  | 10^3^ |  | 10^3^ |
| k_-1,-2_ | time^-1^ | 10^0^ |  | 10^0^ |  |  |  | 10^0^ |  | 10^0^ |
| k_-3,-4_ | time^-1^ | 10^0^ |  | 10^-1^ |  |  |  | 10^0^ |  | 10^-1^ |
| k*_-3,-4_ | time^-1^ | 10^0^ |  | 10^0^ |  |  |  | 10^1^ |  | 10^1^ |
| α_S,T_ | conc.·time^-1^ | 10^2^ |  | 10^2^ |  |  |  | 10^2^ |  | 10^2^ |
| β | time^-1^ | 10^0^ |  | 10^0^ |  |  |  | 10^0^ |  | 10^0^ |
| **Fig 8B,C** | **units** | **TL** | **TC** | **TR** | **ML** | **MC** | **MR** | **BL** | **BC** | **BR** |
| k_1,2,3,4_ | conc.^-1^·time^-1^ | 10^2.5^ |  | 10^2.5^ |  |  |  | 10^2.5^ |  | 10^2.5^ |
| k*_3_,_4_ | conc.^-1^·time^-1^ | 10^2.5^ |  | 10^2.5^ |  |  |  | 10^2.5^ |  | 10^2.5^ |
| k_5_ | time^-1^ | 10^3^ |  | 10^3^ |  |  |  | 10^6^ |  | 10^6^ |
| k_-1,-2,-3,-4_ | time^-1^ | 10^0^ |  | 10^4^ |  |  |  | 10^0^ |  | 10^4^ |
| k*_-3_,_-4_ | time^-1^ | 10^0^ |  | 10^4^ |  |  |  | 10^0^ |  | 10^4^ |
| α_S,T_ | conc.·time^-1^ | 10^2^ |  | 10^2^ |  |  |  | 10^2^ |  | 10^2^ |
| β | time^-1^ | 10^0^ |  | 10^0^ |  |  |  | 10^0^ |  | 10^0^ |
| **Fig 9‡** | **units** | **TL** | **TC** | **TR** | **ML** | **MC** | **MR** | **BL** | **BC** | **BR** |
| k_1,2,3,4_ | conc.^-1^·time^-1^ | 10^2^ |  |  |  |  |  |  |  |  |
| k*_3_,_4_ | conc.^-1^·time^-1^ | 10^2^ |  |  |  |  |  |  |  |  |
| k_5_ | time^-1^ | 10^1.5^ |  |  |  |  |  |  |  |  |
| k_-1,-2,-3,-4_ | time^-1^ | 10^0^ |  |  |  |  |  |  |  |  |
| k*_-3_,_-4_ | time^-1^ | 10^0^ |  |  |  |  |  |  |  |  |
| α_T,S_ | conc.·time^-1^ | 10^2^ |  |  |  |  |  |  |  |  |
| β | time^-1^ | 10^0^ |  |  |  |  |  |  |  |  |
| **Fig 9◊** | **units** | **TL** | **TC** | **TR** | **ML** | **MC** | **MR** | **BL** | **BC** | **BR** |
| k_1,2,3,4_ | conc.^-1^·time^-1^ | 10^2^ |  |  |  |  |  |  |  |  |
| k*_3_,_4_ | conc.^-1^·time^-1^ | 10^2^ |  |  |  |  |  |  |  |  |
| k_5_ | time^-1^ | 10^1.5^ |  |  |  |  |  |  |  |  |
| k_-1,-2,-3,-4_ | time^-1^ | 10^4^ |  |  |  |  |  |  |  |  |
| k*_-3_,_-4_ | time^-1^ | 10^4^ |  |  |  |  |  |  |  |  |
| α_T,S_ | conc.·time^-1^ | 10^2^ |  |  |  |  |  |  |  |  |
| β | time^-1^ | 10^0^ |  |  |  |  |  |  |  |  |
| **Fig 10** | **units** | **TL** | **TC** | **TR** | **ML** | **MC** | **MR** | **BL** | **BC** | **BR** |
| k_1_,_2_,_3_,_4_ | conc.^-1^·time^-1^ | 10^2.5^ |  | 10^2.5^ |  |  |  | 10^2.5^ |  | 10^2.5^ |
| k*_3,4_ | conc.^-1^·time^-1^ | 10^2.5^ |  | 10^2.5^ |  |  |  | 10^2.5^ |  | 10^2.5^ |
| k_5_ | time^-1^ | 10^3^ |  | 10^3^ |  |  |  | 10^3^ |  | 10^3^ |
| k*_5_ | time^-1^ | 0 |  | 0 |  |  |  | 10^3^ |  | 10^3^ |
| k_-1_,_-2_,_-3_,_-4_ | time^-1^ | 10^0^ |  | 10^4^ |  |  |  | 10^0^ |  | 10^4^ |
| k*_-3_,_-4_ | time^-1^ | 10^0^ |  | 10^4^ |  |  |  | 10^0^ |  | 10^4^ |
| α_S_,_T_ | conc.·time^-1^ | 10^2^ |  | 10^2^ |  |  |  | 10^2^ |  | 10^2^ |
| β | time^-1^ | 10^0^ |  | 10^0^ |  |  |  | 10^0^ |  | 10^0^ |

**Supporting Information Table S1. (continued)**

| **Fig 11B** | **units** | **TL** | **TC** | **TR** | **ML** | **MC** | **MR** | **BL** | **BC** | **BR** |
| --- | --- | --- | --- | --- | --- | --- | --- | --- | --- | --- |
| k_1_,_3_ | conc.^-1^·time^-1^ | 10^6^ | 10^-2^ | 10^2^ |  |  |  |  |  |  |
| k_2_,_4_ | conc.^-1^·time^-1^ | 10^-2^ | 10^6^ | 10^2^ |  |  |  |  |  |  |
| k_5_ | time^-1^ | 10^1^ | 10^1^ | 10^1^ |  |  |  |  |  |  |
| k_-1_,_-2_,_-3_,_-4_ | time^-1^ | 0 | 0 | 0 |  |  |  |  |  |  |
| α_S_ | conc.·time^-1^ | 17-83 | 17-83 | 17-83 |  |  |  |  |  |  |
| α_T_ | conc.·time^-1^ | 17-83 | 17-83 | 17-83 |  |  |  |  |  |  |
| α_H_ | conc.·time^-1^ | 10^2^ | 10^2^ | 10^2^ |  |  |  |  |  |  |
| β | time^-1^ | 10^0^ | 10^0^ | 10^0^ |  |  |  |  |  |  |
| **Fig 12B£** | **units** | **TL** | **TC** | **TR** | **ML** | **MC** | **MR** | **BL** | **BC** | **BR** |
| k_1,2,3,4_ | conc.^-1^·time^-1^ | 10^2.5^ |  |  |  |  |  |  |  |  |
| k*_3_,_4_ | conc.^-1^·time^-1^ | 10^2.5^ |  |  |  |  |  |  |  |  |
| k_5_ | time^-1^ | 10^1.5^ |  |  |  |  |  |  |  |  |
| k_-1,-2,-3,-4_ | time^-1^ | 10^1^ |  |  |  |  |  |  |  |  |
| k*_-3_,_-4_ | time^-1^ | 10^1^ |  |  |  |  |  |  |  |  |
| α_S1_ | conc.·time^-1^ | 10^2^-10^4^ |  |  |  |  |  |  |  |  |
| α_T1,S2,T2_ | conc.·time^-1^ | 10^2^ |  |  |  |  |  |  |  |  |
| β | time^-1^ | 10^0^ |  |  |  |  |  |  |  |  |
| **Fig 13**¶ | **units** | **TL** | **TC** | **TR** | **ML** | **MC** | **MR** | **BL** | **BC** | **BR** |
| k_1,4_ | conc.^-1^·time^-1^ | 10^2.5^ |  | 10^2.5^ | 10^3.5^ |  | 10^3.5^ | 10^1.5^ |  | 10^1.5^ |
| k_2,3_ | conc.^-1^·time^-1^ | 10^2.5^ |  | 10^2.5^ | 10^1.5^ |  | 10^1.5^ | 10^3.5^ |  | 10^3.5^ |
| k*_3_ | conc.^-1^·time^-1^ | 10^2.5^ |  | 10^2.5^ | 10^1.5^ |  | 10^1.5^ | 10^3.5^ |  | 10^3.5^ |
| k*_4_ | conc.^-1^·time^-1^ | 10^2.5^ |  | 10^2.5^ | 10^3.5^ |  | 10^3.5^ | 10^1.5^ |  | 10^1.5^ |
| k_5_ | time^-1^ | 10^3^ |  | 10^3^ | 10^3^ |  | 10^3^ | 10^3^ |  | 10^3^ |
| k_-1_,_-4_ | time^-1^ | 10^0^ |  | 10^0^ | 10^-1^ |  | 10^-1^ | 10^1^ |  | 10^1^ |
| k_-2_,_-3_ | time^-1^ | 10^0^ |  | 10^0^ | 10^1^ |  | 10^1^ | 10^-1^ |  | 10^-1^ |
| k*_-3_ | time^-1^ | 10^0^ |  | 10^0^ | 10^1^ |  | 10^1^ | 10^-1^ |  | 10^-1^ |
| k*_-4_ | time^-1^ | 10^0^ |  | 10^0^ | 10^-1^ |  | 10^-1^ | 10^1^ |  | 10^1^ |
| α_S1,T1_ | conc.·time^-1^ | 10^2^ |  | 10^2^ | 10^2^ |  | 10^2^ | 10^2^ |  | 10^2^ |
| α_T non-cog_. | conc.·time^-1^ | 0 |  | 10^4^ | 0 |  | 10^4^ | 0 |  | 10^4^ |
| β | time^-1^ | 10^0^ |  | 10^0^ | 10^0^ |  | 10^0^ | 10^0^ |  | 10^0^ |
| **Fig 13**¥ | **units** | **TL** | **TC** | **TR** | **ML** | **MC** | **MR** | **BL** | **BC** | **BR** |
| k_1,4_ | conc.^-1^·time^-1^ | 10^2.5^ |  | 10^2.5^ | 10^3.5^ |  | 10^3.5^ | 10^1.5^ |  | 10^1.5^ |
| k_2,3_ | conc.^-1^·time^-1^ | 10^2.5^ |  | 10^2.5^ | 10^1.5^ |  | 10^1.5^ | 10^3.5^ |  | 10^3.5^ |
| k*_3_ | conc.^-1^·time^-1^ | 10^2.5^ |  | 10^2.5^ | 10^1.5^ |  | 10^1.5^ | 10^3.5^ |  | 10^3.5^ |
| k*_4_ | conc.^-1^·time^-1^ | 10^2.5^ |  | 10^2.5^ | 10^3.5^ |  | 10^3.5^ | 10^1.5^ |  | 10^1.5^ |
| k_5_ | time^-1^ | 10^3^ |  | 10^3^ | 10^3^ |  | 10^3^ | 10^3^ |  | 10^3^ |
| k_-1_,_-4_ | time^-1^ | 10^2^ |  | 10^2^ | 10^1^ |  | 10^1^ | 10^3^ |  | 10^3^ |
| k_-2_,_-3_ | time^-1^ | 10^2^ |  | 10^2^ | 10^3^ |  | 10^3^ | 10^1^ |  | 10^1^ |
| k*_-3_ | time^-1^ | 10^2^ |  | 10^2^ | 10^3^ |  | 10^3^ | 10^1^ |  | 10^1^ |
| k*_-4_ | time^-1^ | 10^2^ |  | 10^2^ | 10^1^ |  | 10^1^ | 10^3^ |  | 10^3^ |
| α_S1,T1_ | conc.·time^-1^ | 10^2^ |  | 10^2^ | 10^2^ |  | 10^2^ | 10^2^ |  | 10^2^ |
| α_T non-cog_. | conc.·time^-1^ | 0 |  | 10^4^ | 0 |  | 10^4^ | 0 |  | 10^4^ |
| β | time^-1^ | 10^0^ |  | 10^0^ | 10^0^ |  | 10^0^ | 10^0^ |  | 10^0^ |

**Supporting Information Table S1. (continued)**

| **Fig 13**§ | **units** | **TL** | **TC** | **TR** | **ML** | **MC** | **MR** | **BL** | **BC** | **BR** |
| --- | --- | --- | --- | --- | --- | --- | --- | --- | --- | --- |
| k_1,4_ | conc.^-1^·time^-1^ | 10^2.5^ |  | 10^2.5^ | 10^3.5^ |  | 10^3.5^ | 10^1.5^ |  | 10^1.5^ |
| k_2,3_ | conc.^-1^·time^-1^ | 10^2.5^ |  | 10^2.5^ | 10^1.5^ |  | 10^1.5^ | 10^3.5^ |  | 10^3.5^ |
| k*_3_ | conc.^-1^·time^-1^ | 10^2.5^ |  | 10^2.5^ | 10^1.5^ |  | 10^1.5^ | 10^3.5^ |  | 10^3.5^ |
| k*_4_ | conc.^-1^·time^-1^ | 10^2.5^ |  | 10^2.5^ | 10^3.5^ |  | 10^3.5^ | 10^1.5^ |  | 10^1.5^ |
| k_5_ | time^-1^ | 10^3^ |  | 10^3^ | 10^3^ |  | 10^3^ | 10^3^ |  | 10^3^ |
| k_-1_,_-4_ | time^-1^ | 10^0^ |  | 10^0^ | 10^-1^ |  | 10^-1^ | 10^1^ |  | 10^1^ |
| k_-2_,_-3_ | time^-1^ | 10^0^ |  | 10^0^ | 10^1^ |  | 10^1^ | 10^-1^ |  | 10^-1^ |
| k*_-3_ | time^-1^ | 10^2^ |  | 10^2^ | 10^3^ |  | 10^3^ | 10^1^ |  | 10^1^ |
| k*_-4_ | time^-1^ | 10^2^ |  | 10^2^ | 10^1^ |  | 10^1^ | 10^3^ |  | 10^3^ |
| α_S1,T1_ | conc.·time^-1^ | 10^2^ |  | 10^2^ | 10^2^ |  | 10^2^ | 10^2^ |  | 10^2^ |
| α_T non-cog_. | conc.·time^-1^ | 0 |  | 10^4^ | 0 |  | 10^4^ | 0 |  | 10^4^ |
| β | time^-1^ | 10^0^ |  | 10^0^ | 10^0^ |  | 10^0^ | 10^0^ |  | 10^0^ |
| **Fig S1** | **units** | **TL** | **TC** | **TR** | **ML** | **MC** | **MR** | **BL** | **BC** | **BR** |
| k_1_,_2_ | conc.^-1^·time^-1^ |  |  |  |  |  |  | 10^2.5^ | 10^0.5^ | 10^2.5^ |
| k_3_,_4_ | conc.^-1^·time^-1^ |  |  |  |  |  |  | 10^2.5^ | 10^4.5^ | 10^2.5^ |
| k_5_ | time^-1^ |  |  |  |  |  |  | 10^3^ | 10^3^ | 10^3^ |
| k_-1_,_-2_,_-3_,_-4_ | time^-1^ |  |  |  |  |  |  | 0 | 0 | 10^4^ |
| α_S_,_T_ | conc.·time^-1^ |  |  |  |  |  |  | 10^2^ | 10^2^ | 10^2^ |
| β_S free (blk)_ | time^-1^ |  |  |  |  |  |  | 10^0^ | 10^0^ | 10^0^ |
| β_S free (red)_ | time^-1^ |  |  |  |  |  |  | 10^1^ | 10^1^ | 10^1^ |
| β | time^-1^ |  |  |  |  |  |  | 10^0^ | 10^0^ | 10^0^ |
